# An Analysis of the Effect of ZIF-8 Addition on the Separation Properties of Polysulfone at Various Temperatures

**DOI:** 10.3390/membranes11060427

**Published:** 2021-06-04

**Authors:** Kseniya Papchenko, Giulio Risaliti, Matteo Ferroni, Meganne Christian, Maria Grazia De Angelis

**Affiliations:** 1 Department of Civil, Chemical Environmental and Materials Engineering, DICAM, University of Bologna, Via Terracini 28, 40131 Bologna, Italy; kseniya.papchenko2@unibo.it (K.P.); giulio.risaliti@studio.unibo.it (G.R.); 2 CNR-IMM Section of Bologna, Via Gobetti 101, 40129 Bologna, Italy; ferroni@bo.imm.cnr.it (M.F.); christian@bo.imm.cnr.it (M.C.); 3 Department of Civil, Environmental, Architectural Engineering and Mathematics, Università degli Studi di Brescia, Via Valotti, 9, 25123 Brescia, Italy; 4 Institute for Materials and Processes, School of Engineering, University of Edinburgh, Sanderson Building, Robert Stevenson Road, Edinburgh EH9 3FB, UK

**Keywords:** gas separation, CO_2_ capture, mixed matrix membranes

## Abstract

The transport of H_2_, He, CO_2_, O_2_, CH_4_, and N_2_ at three temperatures up to 65 °C was measured in dense, thick composite films formed by amorphous Polysulfone (PSf) and particles of the size-selective zeolitic imidazolate framework 8 (ZIF-8) at loadings up to 16 wt%. The morphological and structural properties of the membranes were analyzed via SEM and density measurement. The addition of ZIF-8 to PSf enhances the H_2_ and He permeabilities up to 480% with respect to the pure polymer, while the ideal H_2_/CO_2_ and He/CO_2_ selectivities of MMMs reach values up to 30–40% higher than those of pure PSf. The relative permeability and diffusivity enhancements are higher than those obtained in other polymers, such as PPO, with the same amount of filler. The Maxwell–Wagner–Sillars model is able to represent the MMM H_2_/CO_2_ separation performance for filler volume fractions below 10%.

## 1. Introduction

In the 40 years since the first industrial gas separation membrane systems were installed, the technology has grown into an industry with system sales approaching one billion dollars. Membranes are currently used with major successes in four applications that represent 80−90% of the membrane market: nitrogen production, natural gas treatment, hydrogen recovery, and vapor recovery [1,2,3]. Currently, Polysulfone remains one of the most widely used materials for hydrogen recovery, characterized by good permeances and acceptable selectivities [4,5,6].

The separation of hydrogen/CO_2_ mixtures is a common problem in refineries and petrochemical plants and represents a necessary step in a number of industrial processes. For example, steam methane reformers are commonly used in refineries, and CO_2_ removal from H_2_/CO_2_ mixture allows both to produce hydrogen and to decarbonize fuel feeds before a secondary combustion step to generate power for electricity. Other hydrogen separations involve adjusting molar ratios of syngas (H_2_/CO), hydrogen recovery in refinery hydrotreaters (H_2_/CH_4_), and ammonia purge gas recovering (H_2_/N_2_) [1,2,7]. Though these applications are mostly solved problems, membranes with increased selectivity at the same permeance can reduce the compressor size, and membranes with increased permeance at the same selectivity can reduce the membrane areas required, thus, leading to a potential reduction in the total process cost of 5−10% [3,7].

Criteria for selecting membranes for a given application are complex. Nonetheless, durability, mechanical integrity at the operating conditions, productivity, and separation efficiency are important stipulations that must be balanced against cost in all cases [3]. Polysulfone has an important role in membrane separation technology thanks to good mechanical and thermal properties, good resistance to plasticization, good processability, and relatively low-cost. Therefore, it was chosen in the present work for a further investigation of an improvement of its gas transport properties, with particular regard to the separation of H_2_ and He from CO_2_.

While the most important advantages of polymeric membranes are easy fabrication and scalability, they suffer from an intrinsic trade-off between permeability and selectivity, as described by Robeson and Freeman [8,9,10]. Moreover, this trade-off relationship results to be dependent on temperature [11]. Thus, a constant research for improvement characterizes today’s gas separation field. One possible solution is represented by the combination of two different classes of materials properties into composite membranes. Mixed matrix membranes (MMMs) indeed combine an inorganic or inorganic–organic hybrid material in the form of micro- or nanoparticles with a continuous polymeric phase.

Currently, one of the most interesting choices for MMMs production consists in using metal organic frameworks (MOFs) as dispersed phase. The organic nature of the linkers that connect metal clusters to one another in these materials offers an intrinsically better compatibility with polymers with respect to purely inorganic particles and potentially infinite design choices [12,13]. However, pure MOF membranes are not able to reach the expected high selectivities because of imperfections, such as pinholes and cracks, thus, opting for MOF dispersion in the polymeric matrix is preferable [14]. Several crucial aspects should be highlighted when considering MMMs fabrication: firstly, good interfacial adhesion between the two phases must be achieved, to prevent the formation of non-selective voids at the interface; secondly, gas diffusion into the filler pores must not be blocked [15,16,17].

Zeolitic imidazole frameworks (ZIFs) belong to a particular class of MOFs that presents an isomorphism with zeolites. Similar to all MOFs, ZIFs provide a wide range of configurations that can be obtained by changing the imidazolate/imidazolate-like linkers and the coordination metal. This feature leads to different topologies and dimensions of the pores. ZIF-8 is a well-known ZIFs family member with molecular sieving properties [18] and commercial availability. Thus, ZIF-8 is a potentially successful choice as a filler for membrane properties enhancement.

Different MMMs have already been analyzed, based on a variety of polymers and MOFs [11,19,20,21,22,23,24,25], and various reviews are already available on the topic [26,27,28,29,30]. In addition, several attempts were already made in order to enhance Polysulfone gas transport properties. The addition of different MOFs, such as MIL-101(Cr) [31], Bio-MOF-1 [32], Cu_3_(BTC)_2_ [33], and MIL-53(Al) [34], was shown to produce a beneficial effect in terms of gas permeabilities and selectivities. Different groups studied and reported the positive effect of ZIF-8 addition into Polysulfone hollow fibers, flat asymmetric or dense membranes [35,36,37,38,39,40,41,42]. However, most studies refer to a narrow set of analyzed gases, filler loadings, and test temperatures.

In this work PSf/ZIF-8 membranes were prepared via solvent casting at three different ZIF-8 loadings, reaching 16% by weight of filler. The permeability and diffusivity of six different gases, H_2_, He, O_2_, N_2_, CO_2_, and CH_4_, were explored at three different temperatures, 35 °C, 50 °C, and 65 °C, to allow the analysis of thermal behavior and the calculation of the activation energies for the various processes. The quality of the composite films obtained was also analyzed via SEM and density analysis.

This systematic study of the effect of the addition of ZIF-8 on Polysulfone, including the influence of different variables such as temperature, filler loading, gas molecular size and nature, allows to fill the gaps currently existing in the knowledge of the behavior of this mixed matrix membrane.

## 2. Materials and Methods

### 2.1. Materials

**Figure 1:**
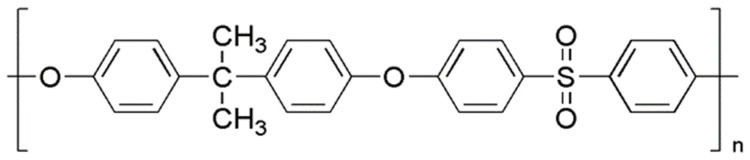
Chemical structure of Polysulfone.

Poly[oxy-1,4-phenylensulfonyl-1,4-phenylenoxy-1,4-phenylen(1-methylethyliden)-1,4-phenylen], known by the trade name of poly(bisphenol-A sulfone) or PSf (chemical structure shown in Figure 1), was purchased in pellets from Sigma Aldrich (St. Louis, MO, USA) and used as received. Polymer weight average molecular weight *M*_W_ and polydispersity index PDI are ~35,000 and 2.2, respectively, as indicated by the vendor.

Polysulfone is a glassy polymer with good gas permeabilities and acceptable selectivities [4,5,6]. As reported by Aitken et al. [5], Bisphenol A base Polysulfone has a relatively high glass transition temperature (186 °C), relatively high density (1.24 g/cm^3^), and a fractional free volume of 0.156.

The commercial sieve selected to produce mixed matrix membranes was ZIF-8 (Basolite^®^ Z1200, Cat. 691348 produced by BASF, Ludwigshafen, Germany). The particle size information for ZIF-8 is provided by the company, and it has a D50 of 4.90 µm.

The solvent used for membrane production was chloroform (CHCl_3_), and it was purchased from Sigma Aldrich and used as received. Gases used for permeation tests, oxygen (O_2_), nitrogen (N_2_), helium (He), hydrogen (H_2_), methane (CH_4_), and carbon dioxide (CO_2_), were purchased from Fluido Tecnica (Campi Bisenzio, Firenze, Italy) with purities ≥99.5%.

### 2.2. Membrane Preparation

#### 2.2.1. Membrane Casting

Pure polymer and mixed matrix membranes were prepared through the solution casting technique. The solvent used for membrane preparation is chloroform (CHCl_3_), a lower-boiling solvent with respect to those traditionally used for PSf membranes, such as DMF, THF, NMF, and others. The choice of using a low-boiling solvent was motivated by the need to make the film evaporation faster, so as to reduce the possibility of ZIF-8 particles to aggregate but also to make the removal of the solvent easier after the casting procedure. A similar methodology to the one developed by Burmann et al. [40] was followed for MMMs preparation.

A polymer solution at 10 wt% was obtained by dissolving 497.3 mg of PSf in 3 mL of CHCl_3_; the complete dissolution of the polymer in the solvent was obtained through vigorous magnetic stirring at room temperature for at least 24 h. MMMs at three different loadings of filler concentration (filler/(filler+polymer)) were prepared; in particular, membranes at 2 wt%, 8 wt%, and 16 wt% of ZIF-8 were obtained. ZIF-8 was thermally activated at 200 °C under vacuum overnight prior to use in order to remove any impurities that might occlude crystalline cages of the material. Filler solutions were obtained through dissolution of an amount equivalent to the desired final loading of the membrane in 3 mL of CHCl_3_. After an initial magnetic stirring, the uniform dispersion of the sieve in the solvent was promoted by ultrasonication for at least 1h. Then, polymer solution was added to the ZIF-8 solution in three separate fractions (1/6, 2/6, and 3/6) while stirring at intervals of at least 2 h each. Fractionate mixing should reduce the shear stress on ZIF-8 particles, thus avoiding particle deformation while maintaining good dispersion.

After complete mixing was achieved, the final solutions were maintained under magnetic stirring overnight prior to casting. Every suspension was poured onto a petri dish (D50), covered with an aluminium sheet, and left to evaporate at room temperature under a hood. All the obtained membranes showed a homogeneous filler dispersion and their thickness ranged between 20 and 50 µm.

#### 2.2.2. Thermal Treatment

All membranes were subject to thermal annealing under vacuum in order to remove the residual solvent and stabilize the gas transport properties over time. An optimization of the treatment temperature for the removal of solvent was carried out by correlating the time stability of the membrane permeability to the treatment time and temperature.

The final treatment consisted of three steps: the temperature was set at 60 °C for 1 h, then at 100 °C for 2 h, and at last, it was kept at 150 °C for at least 20 h. A progressive increase in temperature is needed to avoid the foaming of the membrane caused by fast solvent evaporation. The PSf glass transition temperature, equal to 186 °C [4], was not reached to avoid filler aggregation caused by higher polymer chain mobility during the treatment.

The performed thermal treatment allowed to achieve time-independent permeability properties, which testify the complete removal of solvent. An incomplete removal would, indeed, result in the permeability of the sample varying with time due to progressive evaporation of the solvent in the membrane during the tests [43,44].

### 2.3. Membrane Characterization

#### 2.3.1. Morphological Characterization

The freeze-fractured samples were mounted over a standard adhesive support for the observation with a ZEISS EVO LS 10 (Carl Zeiss NTS, Oberkochen, Germany) environmental SEM. The operation of the SEM in low vacuum condition (0.1–0.01 mbar pressure range) prevented the electrostatic charging of the sample and allowed the observation of the sample morphology in its pristine condition. The 20 keV beam energy allowed the acquisition of morphological images with the backscattered electrons detector, featuring also significant sensitivity to changes in elemental composition, in order to highlight the dispersion of ZIF-8 particles in the polymeric matrix.

#### 2.3.2. Density Measurement

The determination of the density of the membranes, $\rho_{MMM}$, was performed by means of the buoyancy method, based on the Archimedes’ principle, using a density kit (MS-DNY-54) on a high precision balance (Mettler Toledo, NewClassic MF MS105DU). Deionized water was used to determine the hydrostatic weight of the sample. A wetting agent (Pervitro 75% 72409) was used to avoid the formation of air bubbles on the submerged film, which might affect the measurements, introducing a negligible change in the water density. The temperature of the fluid was monitored with a thermometer (±0.1 °C) to determine the proper water density, $\rho_{H_{2}O}$, and in order to calculate the sample density as follows:

(1) $$\rho_{MMM}=\frac{m_{MMM}^{Air}}{\left(m_{MMM}^{Air}-m_{MMM}^{H_{2}O}\right)}\rho_{H_{2}O}\left(T\right)$$

where $m_{MMM}^{Air}$ is the weight of the sample measured in air, while $m_{MMM}^{H_{2}O}$ is the weight measured when the sample was soaked in water. The error associated to weight change due to limited water sorption by Polysulfone was considered, together with water density error due to temperature determination.

#### 2.3.3. Gas Permeation Analysis

Pure gas permeability of He, H_2_, O_2_, N_2_, CH_4_, and CO_2_ was evaluated at different temperatures (35 °C, 50 °C, and 65 °C) for pure PSf and MMMs at different loadings of the filler, up to 16 wt% of ZIF-8. The fixed-volume, variable-pressure manometric technique and the equipment set-up were schematically represented and described elsewhere [19]. Each permeability experiment was performed at an absolute upstream pressure of ~1.3 bar and initial vacuum condition on the permeate membrane side, thus, pressure was conveniently low to neglect any plasticization effect in the polymeric matrix. The temperature effect was investigated by repeating permeability tests for six gases on the same sample. To ensure that no changes occurred during test performance, at the end of high temperature (65 °C) runs, the temperature was set back at 35 °C, and helium permeability was measured again at the initial test conditions. All samples showed thermal stability, expressed through stable permeability results after a full temperature cycle.

The permeability of the component *i*, ${\mathbb{P}}_{i}$, is defined as the ratio between the molar flux $J_{i}$ across the membrane and the partial pressure gradient across the membrane of thickness *l*, at steady state:

(2) $${\mathbb{P}}_{i}=J_{i}\frac{l}{p_{i,\;feed}-p_{i,\;\;perm}}$$

If the Fick’s law governs the diffusion in the membrane, and phase equilibrium is attained at the gas–membrane interface, the solution-diffusion model is obeyed [45], and the permeability coefficient can be split into two factors: the diffusion coefficient, $D_{i}$, a predominantly kinetic factor that measures the mobility of different species in the polymeric matrix, and the sorption coefficient, $S_{i}$, a thermodynamic factor reflecting the solubility of different species in the polymer:

(3) $${\mathbb{P}}_{i}=D_{i}S_{i}$$

Caution is needed when using this approach, valid for dense homogenous polymers, on mixed matrix membranes containing porous fillers. This is particularly true when the above formula is used to estimate properties, such as the solubility coefficient, from measured values of diffusivity and permeability, e.g., from a permeation experiment.

The ideal selectivity can provide an indication of the ability of the membrane to separate two gases and can be evaluated as the ratio between pure gas permeabilities. Under the assumption of solution-diffusion model and negligible downstream pressure, the ideal selectivity can be conveniently split into diffusivity selectivity, $\alpha^{D}$, and solubility selectivity, $\alpha^{S}$, as follows:

(4) $$\alpha_{ij}=\frac{{\mathbb{P}}_{i}}{{\mathbb{P}}_{j}}=\frac{D_{i}S_{i}}{D_{j}S_{j}}=\alpha_{ij}^{D}\alpha_{ij}^{S}\;$$

The time-lag, $\theta_{L}$, is the characteristic time of gas molecules diffusing through the polymer and can be evaluated in a closed-volume system as the one used in the present study [45,46]. Considering zero initial concentration of gas across the membrane, the time-lag can be related to the membrane thickness and gas diffusivity as follows:

(5) $$D=\frac{l^{2}}{6\theta_{L}}$$

The dependence of the transport and sorption properties on temperature can be used to evaluate the membrane performance at temperatures different from those investigated experimentally and to enable process calculations in real conditions. Permeability, diffusivity, and solubility dependence on temperature can be described by Arrhenius-like equations [47] as follows:

(6) $$\mathbb{P}={\mathbb{P}}_{\infty }exp\left(-\frac{E_{\mathbb{P}}}{RT}\right)$$

(7) $$D=D_{\infty }exp\left(-\frac{E_{D}}{RT}\right)$$

(8) $$S=S_{\infty }exp\left(-\frac{\Delta H_{S}}{RT}\right)$$

Here, $E_{\mathbb{P}}$ and $E_{D}$ are the activation energies of the permeation and diffusion processes, respectively, while ${\mathbb{P}}_{\infty }$, $D_{\infty }$, and $S_{\infty }$ are the temperature independent pre-exponential terms that represent the permeation, diffusion, and sorption coefficients at infinite temperature. The heat of sorption, $\Delta H_{S}$, defines the thermal energy associated to gas sorption. Diffusion is a thermally activated process characterized by values increasing with temperature due to enhanced mobility of the penetrant and to increased chain flexibility of the polymer. Under the assumption of solution-diffusion model the heat of sorption can be simply estimated as the difference between $E_{\mathbb{P}}$ and $E_{D}$. Such quantity is usually dominated by the condensation of the gas into the membrane, rather than by the mixing between gas and polymer molecules and is, thus, usually negative.

## 3. Results and Discussion

### 3.1. Membrane Appearance and Morphology

**Figure 2:**
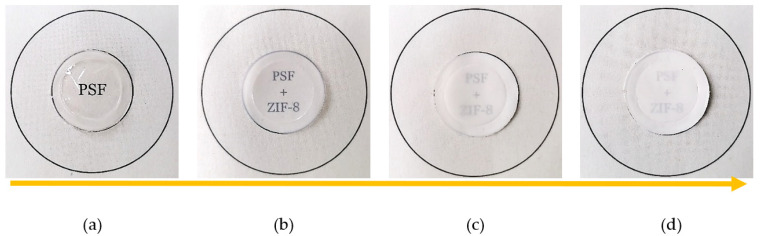
Membrane samples used for permeation tests: (**a**) PSf; (**b**) PSf/2% ZIF-8; (**c**) PSf/8% ZIF-8; (**d**) PSf/16% ZIF-8. The arrow indicates increasing filler content.

The PSf-based mixed matrix membranes at different loadings of ZIF-8 were prepared following the optimized protocol described above. Figure 2 clearly shows that MMMs with ZIF-8 are less transparent with respect to pure polymer because of the filler presence. All the prepared membranes were homogeneous macroscopically and present a good flexibility.

All membranes resisted the pressure difference applied across the membrane during permeation tests without pinholes or cracks formation.

#### 3.1.1. SEM Analysis

**Figure 3:**
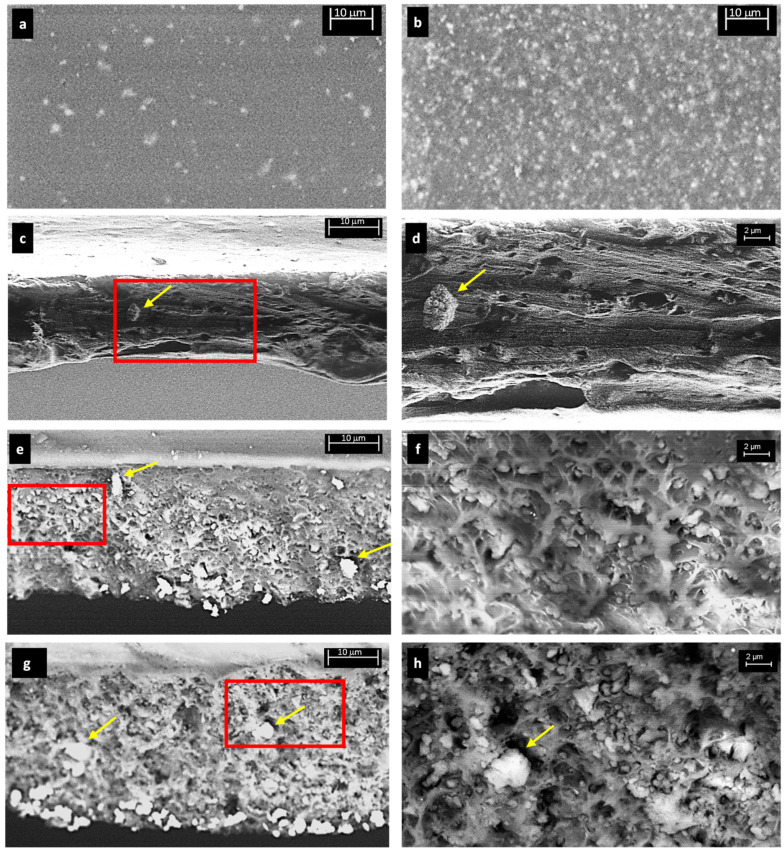
SEM images of the MMMS inspected in this work. Surface of films of PSF/2wt%ZIF-8 (**a**) and PSF/16wt%ZIF-8 (**b**). Cross section of films of PSF/4wt%ZIF-8 (**c**), PSF/8wt%ZIF-8 (**e**), and PSF/16wt%ZIF-8 (**g**). Red rectangles indicate the portion of film inspected in Figure d,f,h. Magnified views of the cross section of films of PSF/4wt%ZIF-8 (**d**), PSF/8wt%ZIF-8 (**f**), and PSF/16wt%ZIF-8 (**h**). Yellow arrows indicate the larger aggregates of ZIF-8.

The surface morphology of the MMM films is presented in Figure 3a,b for the sample with the lowest and highest filler loading, 2% and 16%, respectively. It can be seen that in both samples the particles were distributed rather uniformly over the film area, with a higher concentration in the case of the sample with the highest loading. To analyze in detail the distribution of filler across the film thickness and the size of the particles, it was necessary to look at the SEM cross-sectional images. These ones are reported in Figure 3c–h for samples containing 4%, 8%, and 16% of ZIF-8. The sample containing 2% of ZIF-8 did not undergo a sharp cut and, therefore, was not considered in the cross-sectional inspection. Figure 3c,d, thus, refer to two different magnifications of the same sample, containing 4% of ZIF-8: in such case, it was not necessary to highlight the elemental composition as the particles were very evident; in particular, in Figure 3d, one could notice many small black spots that represented small particle sites and a larger aggregate on the left. In Figure 3e–h, relative to samples with higher loadings, the backscattered electrons detector allowed us to reveal with a brighter colour the ZIF-8 particles inside the matrix. The cross-section of such samples became more corrugated and rougher with increasing amounts of filler as the polymer structure was increasingly disrupted by the presence of ZIF particles. Figure 3e,f refer to the sample containing 8% of ZIF-8, and it could be seen that the particles were distributed across all the thickness of the film. Small and larger particles were both visible in this sample. In the sample containing 16% of ZIF-8, depicted in Figure 3g,h, one could notice an ubiquitous distribution of small and large particles in the film thickness.

#### 3.1.2. Density

The density values of pure materials and composite membranes were measured and reported in Table 1 versus filler content. In order to have an idea about the adhesion between the polymer and the filler, one can compare the measured density values to the “ideal” value that would result from additivity between the volume of the pure polymer and of the pure filler, which can be estimated as:

(9) $$\rho_{MMM,\;id}=\frac{\rho_{PSf}\rho_{ZIF-8}}{w_{PSf}\rho_{ZIF-8}+w_{ZIF-8}\rho_{PSf}}$$

The density of ZIF-8 is taken from the literature to be equal to 0.98 g/cm^3^ [19,48].

The comparison between experimental and ideal density values is reported in Table 1. It can be seen that, while the average value of the experimental density was always slightly lower than the value calculated from the additive rule, the difference between the two values never exceeded the experimental uncertainty, which varied between 1 and 1.8% in the various samples. Therefore, the data indicate that samples follow indicatively the volume additivity within the experimental uncertainty. Thus, the presence of voids inside the MMMs was not detectable macroscopically, as it would have resulted in lower-than-additive density values. This result is in agreement with the SEM analysis of the samples that do not show significant voids.

### 3.2. Pure PSf and ZIF-8 Gas Transport Properties

**Figure 4:**
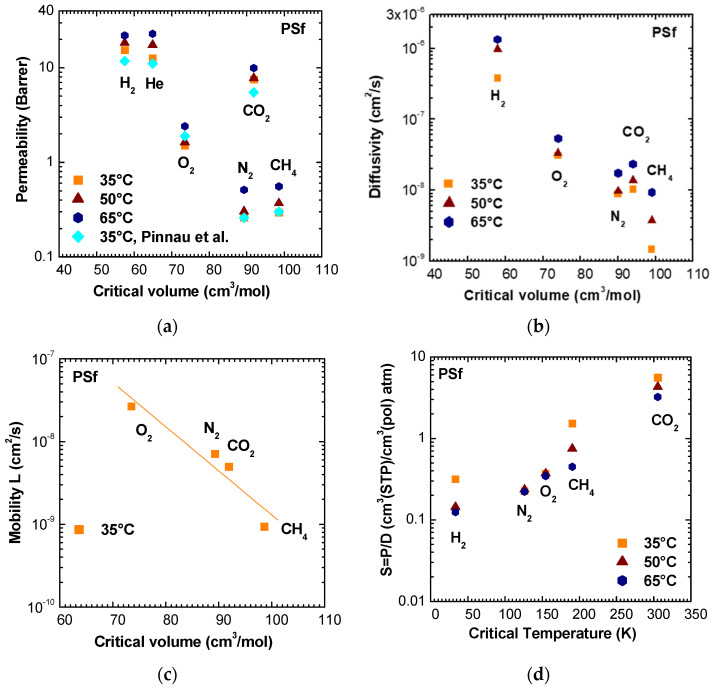
(**a**) Permeability and (**b**) diffusivity of various gases in PSf at 35, 50, and 65 °C as a function of gas critical volume; Permeability data from [50]. (**c**) Mobility of O_2_, CO_2_, N_2_, and CH_4_ at 35 °C, calculated as reported in Section S1.1. (**d**) Solubility coefficient of various gases in PSf at 35, 50, and 65 °C as a function of critical temperature.

An analysis of PSf transport properties was carried on at different temperatures for He, H_2_, CO_2_, O_2_, N_2_, and CH_4_. The results are shown in Figure 4 and it can be seen that the permeability values (Figure 4a) are in agreement with literature data at 35 °C [49,50]. Both permeability (Figure 4a) and diffusivity (Figure 4b) decreased with the penetrant critical volumes, with CO_2_ as the only exception. In this respect, it may be more meaningful to report the mobility, rather than the diffusivity values, because the latter ones are also influenced by thermodynamic factors, which are important in the case of CO_2_. The mobility, on the other hand, is a purely kinetic parameter [51,52,53,54]. A detailed explanation of the calculation procedure of mobility values in this work is reported in Section S1.1 of the Supplementary Information. The mobility values are reported in Figure 4c versus the critical volume and it can be seen that they are in better agreement with critical volume of gases with respect to diffusivity (R^2^(D) = 0.82, R^2^(L) = 0.90).

The solubility coefficient, calculated as the ratio between permeation and diffusion coefficients (Figure 4d), increased with the critical temperature of gases, a parameter that quantified gas condensability, with the only exception given by hydrogen at 35 °C. H_2_ is the most permeable gas in PSf, while CO_2_ is the most soluble, as should be expected due to the higher condensability and polarizability of CO_2_.

Different studies analysed the possibility of using ZIF-8 as a filler in order to improve gas permeability in mixed matrix membranes. From crystallographic data, the smaller window of ZIF-8 was estimated to be equal to 3.4 Å, thus, larger than H_2_ and He effective diameters (2.90 Å and 2.60 Å, respectively). However, ZIF-8 presented a rather flexible structure, and therefore, no sharp molecular sieving took place [18,55]. Indeed, it was shown by measuring diffusivities of molecules of different kinetic diameters that the effective aperture size of ZIF-8 for molecular sieving was more likely to be in the range of 4.0 to 4.2 Å, thus, capable of accommodating molecules of analogous size [18,56].

Table S1 summarizes permeability and ideal selectivity values for the gases considered in this study. Permeability values of PSf and PSf/ZIF-8 membranes are from this work, while ZIF-8 permeability coefficients are indicated as averages values of results reported by different authors [35,48,57,58,59,60,61,62]. Indeed, permeability values in pure ZIF-8 membranes highly depend on the fabrication method and the final thickness of the film, which is often difficult to determine. The H_2_ permeability ranges between 3531 [57] and 10,337 Barrer [58], leading to a high dispersion, reported in Table S1. Selectivity values are quite moderate and present some scatter. Although ZIF-8 permeabilities overcome those of PSf by three or four orders of magnitude, PSf sieving abilities are higher than those of ZIF-8 for almost all gas pairs with the exception of H_2_/CO_2_ and He/CO_2_, which motivates the choice of ZIF-8 to enhance the separation performance of PSf towards these two gas mixtures.

### 3.3. Permeability and Permselectivity

Table S1 reports permeability and ideal selectivity values for six gases in membranes at 0%, 2%, 8%, and 16% by weight ZIF-8 loadings at 35 °C, 50 °C, and 65 °C. The tests were repeated at least three times for every temperature and filler fraction. The overall error was below ±10% for pure PSf and PSf with 2% ZIF-8 loading and below ±15% for the other membranes.

#### 3.3.1. Effect of Filler Loading

**Figure 5:**
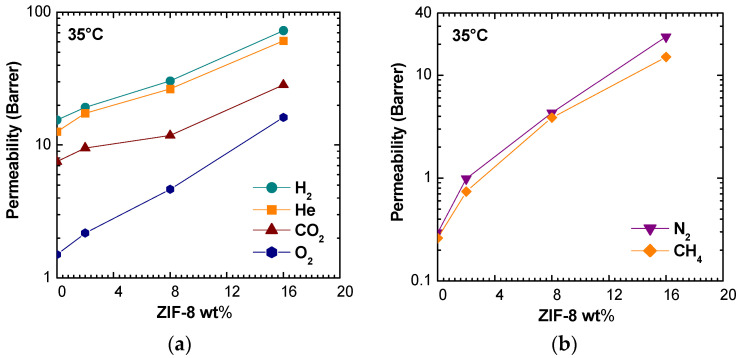
Permeability of various gases in PSf/ZIF-8 mixed matrix membranes as a function of ZIF-8 wt%: (**a**,**b**) at 35 °C.

Pure gas permeability values at 35 °C are shown in Figure 5 for membranes with ZIF-8 weight loadings of 0%, 2%, 8%, and 16%. It can be noted that permeability increased monotonously with filler loading for all gases. The qualitative trend was the same at different temperatures (Figure S1 in Supplementary Material), though the enhancement ratio tended to be lower when temperature was higher. The filler presence affected more the larger gases, N_2_ and CH_4_, enhancing their permeability values by a factor of about 60 and 80, respectively, at 35 °C and of about 20 at 65 °C. O_2_ permeability was enhanced by a factor of 10 at the lowest temperature and by a factor of five at the highest one. The permeability increases for H_2_, He, and CO_2_ were similar and ranged between 5 and 2.5 times in the studied temperature range.

**Figure 6:**
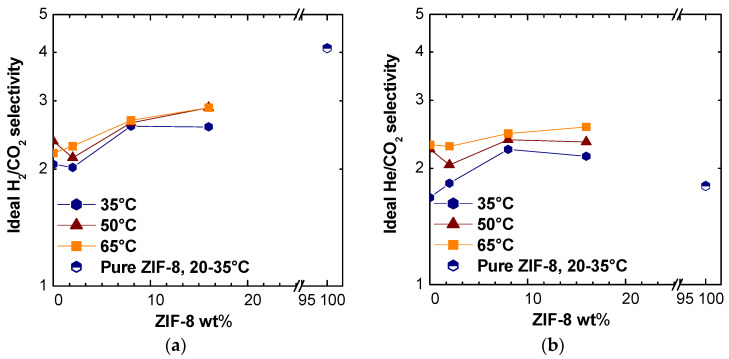
Selectivity in PSf/ZIF-8 mixed matrix membranes as a function of ZIF-8 content: (**a**) H_2_/CO_2_; (**b**) He/CO_2_. For comparison, also, the average value reported for ZIF-8 is included at temperatures between 20 and 35 °C [27,42,43,44,45,46,47,48].

We could observe a moderate increase in ideal selectivity for the H_2_/CO_2_ and He/CO_2_ pairs at different temperatures (Figure 6), while the selectivity for the other couples, as expected due to the intrinsic properties of ZIF-8, decreased. The H_2_ and He permeabilities increased up to 480% with respect to the pure polymer, the selectivity of MMMs reached values up to 30% higher than those of pure PSf. Moreover, the selectivity values for H_2_/CO_2_ seemed to tend to the pure average ZIF-8 value estimated from the literature. For the He/CO_2_ selectivity, the value estimated for pure ZIF-8 seemed lower than those obtained on the mixed matrices in this work, but it must be noticed that this value showed a large variability in the literature [35,48,57,58,59,60,61,62].

The addition of ZIF-8 is expected to worsen pure polymer selectivities for H_2_/CH_4_, CO_2_/CH_4_, O_2_/N_2_ and CO_2_/N_2_ gas pairs, as this filler is less selective than PSf, therefore the behavior reported in Table S1 is qualitatively reasonable. For the samples containing the highest loadings of ZIF-8, the selectivity towards the above-mentioned gas mixtures seems to be lower than that of pure ZIF-8. This behavior could be explained by the presence of some unselective voids in the membranes with higher loadings, a phenomenon which however was ruled out by the density analysis. The modeling analysis carried out in the respective paragraph will allow to gain a deeper understanding of this behavior. It was also worth noticing that CH_4_/N_2_ selectivity values in composite membranes were similar or higher than those of both filler and polymer, reaching values up to 40% higher than those of pure Polysulfone and 75% higher than those of pure ZIF-8, when PSf/16% ZIF-8 membrane was considered.

By looking at the previous results on PSf/ZIF-8 mixed matrix membranes, one can find only one work relative to dense symmetric membranes [40]. Burmann and coworkers indeed analysed the H_2_/CH_4_ and O_2_/N_2_ separation through a spin-coated membrane with 8 wt% ZIF-8 loading in PSf at 35 °C. The mixed matrix membrane obtained had a generally better performance in terms of permeability and size-selectivity with respect to the corresponding one inspected in this work. Indeed, the mixed gas selectivity for the two gas couples decreased after addition of ZIF-8, but to a limited extent. This behaviour can be attributed to the fact that the ZIF-8 particles were smaller and/or better incorporated in the polymer matrix, due also to the spin coating technique, which may have reduced particle aggregation during casting. Furthermore, selectivities were measured in that work in the mixed gas state and not estimated as ideal values like in the present work. Unfortunately, the data referred to only one composition of ZIF-8, and a more comprehensive and systematic comparison was not possible.

Other literature results refer to asymmetric membranes and to a range of loadings that is smaller than the one considered in this work. The paper of Nordin et al. refers to flat asymmetric membranes [38], while the one by Khan et al. [36] deals with hollow fibres. Both authors observe that the CO_2_/CH_4_ separation behaviour is enhanced by addition of ZIF-8 when the loading is small, while a deterioration of the selectivity occurs at higher loadings, similarly to what was observed in this work, due to the larger relative increase of CH_4_ permeability with respect to the CO_2_ one.

#### 3.3.2. Effect of Temperature

**Figure 7:**
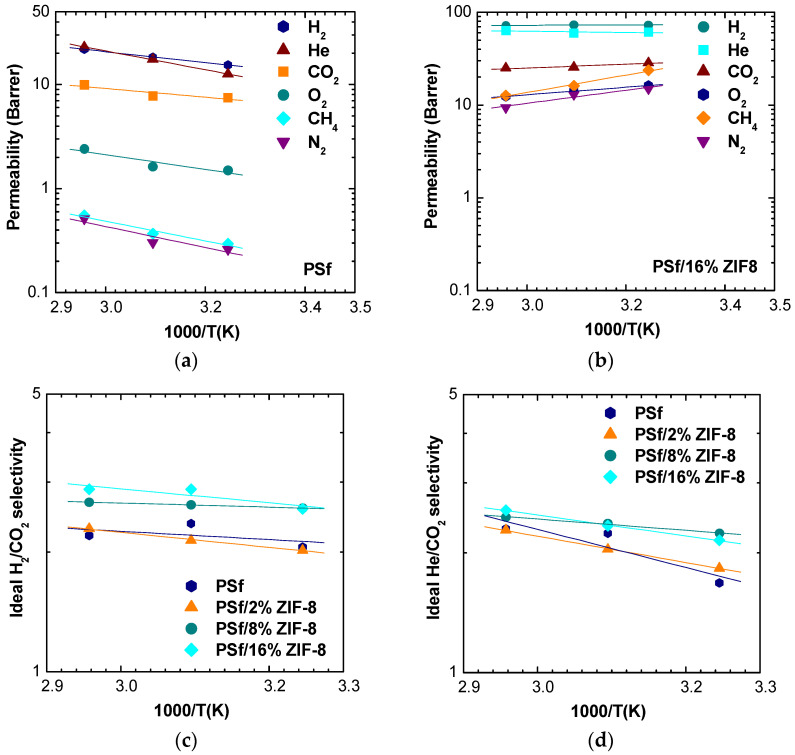
Permeability and selectivity in PSf/ZIF-8 mixed matrix membranes as a function of 1000/T: (**a**) Permeability in PSf; (**b**) Permeability in PSf/16% ZIF-8; (**c**) H_2_/CO_2_ selectivity; and (**d**) He/CO_2_ selectivity in all the MMMs inspected.

The effect of temperature on the transport properties can be investigated by plotting permeability and selected selectivity data as an inverse function of temperature for various filler loadings. In Figure 7a,b, data for pure PSf and PSf/16% ZIF-8 are shown, while data for other loadings are reported in Figure S2.

Permeability values follow the same order at different temperatures and compositions, namely, ${\mathbb{P}}_{H_{2}}≅\;{\mathbb{P}}_{He}>{\mathbb{P}}_{CO_{2}}>{\mathbb{P}}_{O_{2}}>{\mathbb{P}}_{CH_{4}}≅{\mathbb{P}}_{N_{2}}$. At the same time, though, methane and nitrogen permeabilities become comparable to values showed by oxygen at higher ZIF-8 loadings.

All gas permeabilities show a moderate tendency to increase with temperature in pure PSf and PSf/2% ZIF-8 membranes. At higher loading, an inversion of this behavior can be observed for N_2_ and CH_4_. The change in permeability slope with temperature corresponds to the change in sign of activation energy from positive to negative, which will be discussed in the relative section. As far as the selectivities are concerned, one can notice that the dependence on temperature is positive but very weak in the case of the H_2_/CO_2_ couple (Figure 7c). For the He/CO_2_ pair, there is a stronger increase of selectivity with temperature, which, however, tends to diminish at higher filler loadings (Figure 7d).

**Figure 8:**
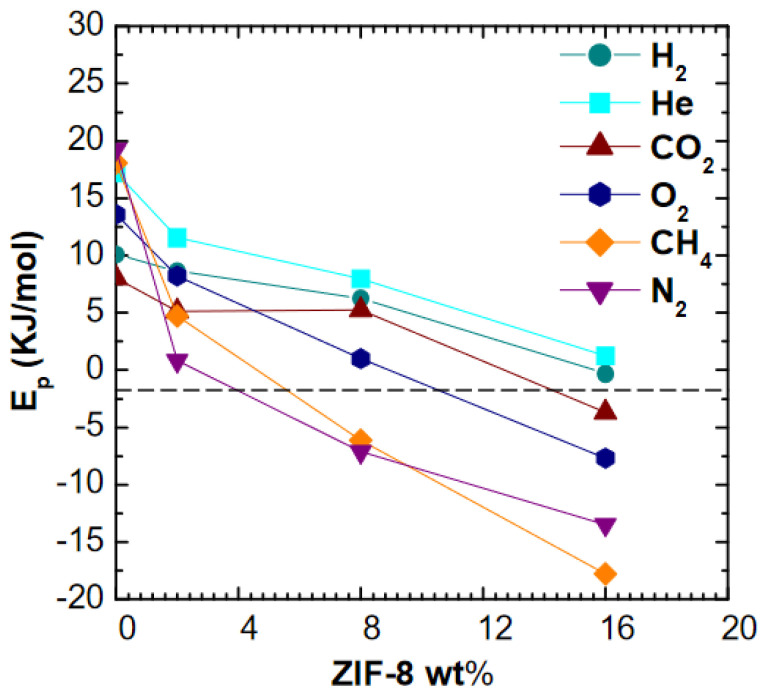
Activation energy of permeation of various gases in PSf/ZIF-8 mixed matrix membranes as a function of ZIF-8 wt%.

It can be seen in Figure 8 that activation energies of permeation decrease rapidly with filler loading, becoming negative for all gases, except for He and H_2_. A similar qualitative effect was observed by Benedetti et al. [19] in the case of ZIF-8 addition to PPO matrix, where higher filler loadings made permeability a weaker function of temperature for He, CO_2_, N_2_, and CH_4_. However, the effect is much more marked in this work, especially for the larger molecules, and indicates that the energetic barrier to gas permeation decreases with increasing filler loading. It must be noticed that the activation energies of permeation of CO_2_ and O_2_ are negative in pure ZIF-8 membrane, while values close to zero are reported for H_2_, He, N_2_, and CH_4_ [63]. Thus, it is qualitatively reasonable that the activation energy of diffusion drops with filler loading: in such conditions the permeation activation energy becomes dominated by the heat of sorption, which is negative due to the condensation effect. Other possible reasons for this behavior, such as the creation of additional free volume that reduces the barrier to diffusion, are also possible although their presence was not detected in appreciable amounts through density tests. A comparison with models performed in the respective section will allow to gain a better understanding of this behavior.

### 3.4. Diffusivity and Diffusivity-Selectivity

Diffusivity values for five gases, namely, H_2_, CO_2_, O_2_, N_2_, and CH_4_, were evaluated using the time-lag technique, as described above. In case of helium, the diffusivity could not be estimated with reasonable accuracy due to small values of time lag. Table S2 lists diffusivity and diffusivity selectivity values for five gases in membranes at 0%, 2%, 8%, and 16% by weight ZIF-8 loadings at 35 °C, 50 °C, and 65 °C. For better clarity, Table S3 reports time lag values for different membrane loadings and for all temperatures.

#### 3.4.1. Effect of Filler Loading

**Figure 9:**
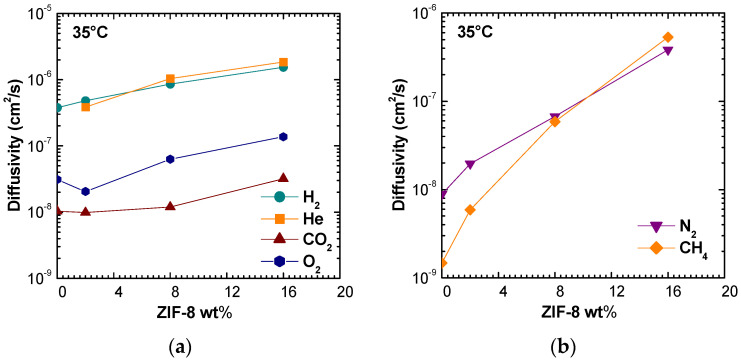
Diffusivity of various gases in PSf/ZIF-8 mixed matrix membranes as a function of ZIF-8 wt%: (**a**,**b**) at 35 °C. The behavior at 50 and 65 °C is reported in Figure S3.

Figure 9 shows the diffusivity values correlated with filler loading at 35 °C. Values obtained at 50 °C and 65 °C are reported in Figure S3. It can be seen that ZIF-8 addition promotes diffusion enhancement for all gases considered. The trend is consistent at all temperatures and the major effect is observed for nitrogen and methane, as for the permeability coefficients. In particular, it can be observed that, for these two gases, the diffusivity tends to approach values close to the order of magnitude (10^−6^ cm^2^/s) reported for pure ZIF-8 [14].

#### 3.4.2. Effect of Temperature

**Figure 10:**
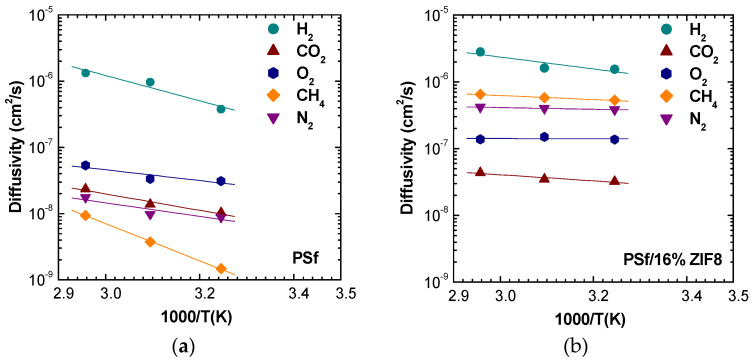
Diffusivity of various gases in PSf/ZIF-8 mixed matrix membranes as a function of 1000/T: (**a**) PSf; (**b**) PSf/16% ZIF-8. The behavior at intermediate loadings is reported in Figure S4.

The analysis of temperature dependence allows us to visualize clearly how the filler addition to the polymeric matrix induces a reduction in temperature effects on diffusion coefficients for all the gases. Figure 10 and Figure S4 show the results of this analysis. In particular, it can be noted that CH_4_ diffusion is highly affected by temperature in pure PSf, changing by one order of magnitude over a 30 °C span. ZIF-8 addition of just 2% by weight lowers this increase by more than half, while the filler presence at 16% makes the diffusion almost independent from temperature.

**Figure 11:**
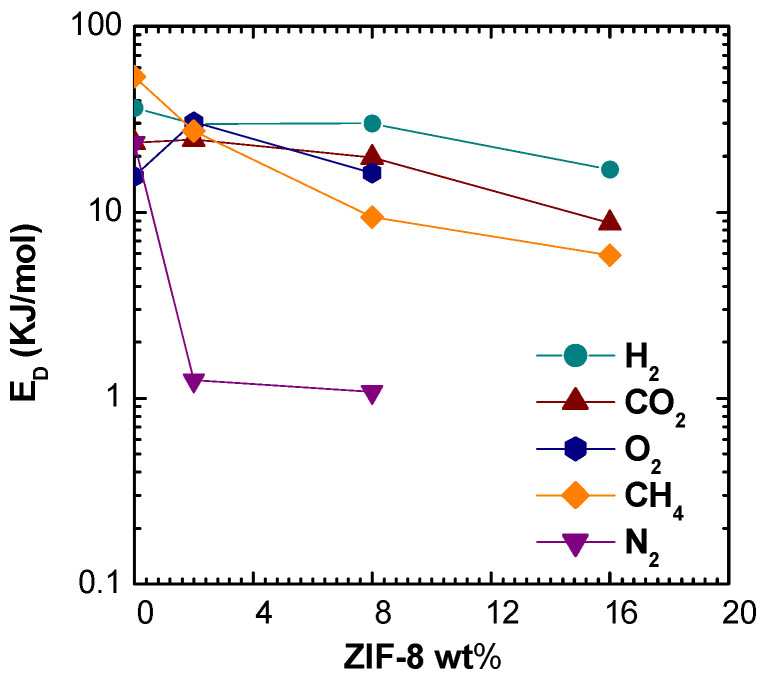
Activation energy of diffusion of various gases in PSf/ZIF-8 mixed matrix membranes as a function of ZIF-8 wt%.

At low filler loadings, the diffusion coefficients are inversely related to the gas critical volume, following the order: $D_{H_{2}}>D_{O_{2}}>D_{CO_{2}}>D_{N_{2}}>D_{CH_{4}}$. However, when a higher filler loading is considered, the trend changes, and the following order is observed: $D_{H_{2}}>D_{CH_{4}}>D_{N_{2}}>D_{O_{2}}>D_{CO_{2}}$. The inversion of diffusivity behavior can be explained by the fact that CH_4_ and N_2_, the larger molecules, experience a huge increase of diffusivity. Such behavior can be due to the fact that such gases diffuse mostly in the ZIF-8 domains, rather than in the polymer phase. This hypothesis is also consistent with the values of activation energies of diffusion, reported in Figure 11. ZIF-8 addition contributes to lower the activation energy of diffusion for all gases, but especially for CH_4_ and N_2_. Therefore, the ZIF-8 presence actively reduces the energetic barrier of a diffusive jump in the membrane material. To some extent, this phenomenon could also be due to the creation of additional free volume areas that enhance diffusion, although such regions were not detected through macroscopic density tests, that are proven to reveal their presence. In the modeling section, a comparison with models for ideal composites will allow to detect the presence of non idealities and gain a better understanding of this behavior.

The reduction of the value of $E_{D}$, combined with the negative values of heat of sorption, leads to the negative values of $E_{\mathbb{P}}$ observed before.

### 3.5. Permeability and Diffusivity Enhancement

**Figure 12:**
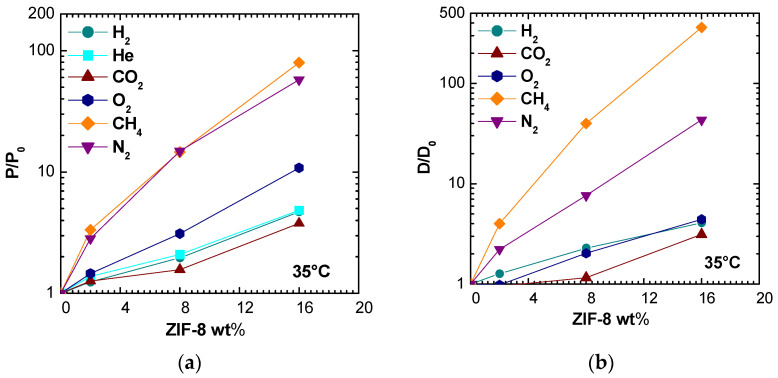
Relative permeability (**a**) and diffusivity (**b**) increase of various gases in PSf/ZIF-8 mixed matrix membranes as a function of ZIF-8 wt% at 35 °C. The behavior at 50 and 65 °C is reported in Figure S5.

In the following section, the gas transport properties enhancement of the resulting MMMs will be evaluated with respect to the pure polymer matrix. Figure 12 and Figure S5 display the permeability enhancements on the right and on the left, the corresponding diffusivity enhancements at all temperatures and for different ZIF-8 loadings. For all gases, except for N_2_ and CH_4_, the raise in diffusivity was lower with respect to increase in the permeability. This behavior may indicate an enhancement due to ZIF-8 addition on both diffusivity and solubility of the gases, if the solution-diffusion model holds true.

**Figure 13:**
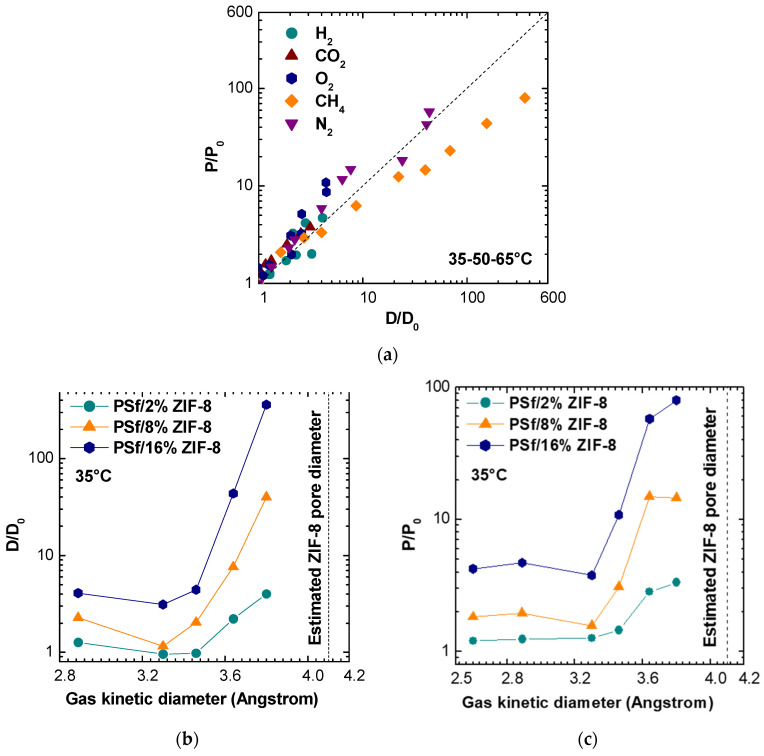
(**a**) Comparison between the permeability and diffusivity enhancement for various gases at different temperatures, observed after addition of variable amounts of ZIF-8 in PSf. (**b**) Diffusivity enhancement versus gas kinetic diameter, for different PSf/ZIF-8 MMMS, at 35 °C. (**c**) Permeability enhancement versus gas kinetic diameter, for different PSf/ZIF-8 MMMS, at 35 °C.

Figure 13 allows for a better correlation of permeability enhancements to diffusivity ones for different gases at different temperatures and filler loadings. When data points are close to the diagonal, permeability is purely governed by diffusivity. When data lie above the diagonal, and if the solution-diffusion model is valid, there is also a beneficial effect of ZIF-8 on the gas solubility, while the opposite is true when the data lie below the line.

Nitrogen permeability seems to depend exclusively on diffusivity, leading to the conclusion that the filler inclusion in PSf matrix enhances mainly the kinetic part of N_2_ transport. For the case of CH_4_, the diffusivity enhancement is higher than the permeability one, especially at higher filler loadings. Indeed, as it can be seen from Figure 13b,c, which report permeability and diffusivity enhancement values at 35 °C versus the gas kinetic diameter for different filler loadings, the permeability and diffusivity of gases with sizes below about 3.4 Å is moderately enhanced by the addition of ZIF-8, while the relative enhancement observes a steep increase when larger molecules are concerned. This seems to confirm the assumption that the ZIF-8 pore diameter is not 3.4 Å, as estimated from XRD analysis, but is more likely to be located between 4.0 and 4.2 Å [18,56]. This estimated value is represented as a dashed vertical line in Figure 13b,c. The data seem to indicate that the addition of ZIF-8 is particularly beneficial for those gases whose size is larger and closer to the value of the ZIF-8 aperture and indicate that such gases find preferential diffusion pathways in ZIF-8 domains. Similar trends to the one reported in Figure 13 were obtained also at 50 and 65 °C and are not reported for the sake of brevity.

### 3.6. Estimated Solubility

**Figure 14:**
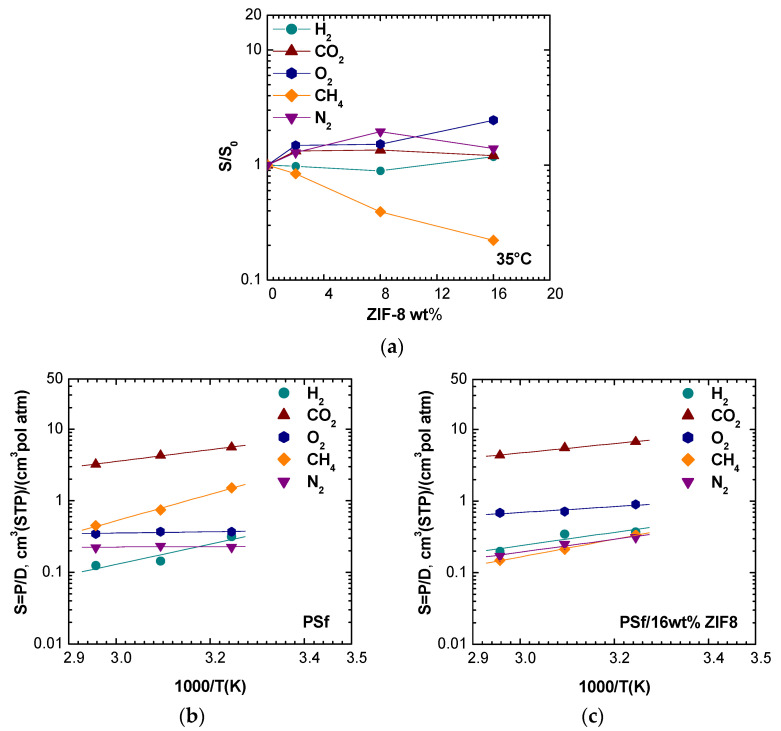
(**a**) Solubility enhancement observed after adding ZIF-8 to PSf for various gases at 35 °C. Solubility versus 1000/T for (**b**) PSf; (**c**) PSf/16% ZIF-8.

The solubility coefficient was calculated as the ratio between permeability and diffusivity, assuming the validity of the solution-diffusion model, which however tends to be less reliable at higher filler loadings [45]. Figure 14a reports the solubility enhancement versus the filler loading at 35 °C. For H_2_, He, O_2_, N_2_, and CO_2_ the solubility was slightly promoted or not clearly affected by the filler presence in almost all cases, while for CH_4_, the solubility apparently decreased. The reason for this behavior could be due to the fact that CH_4_ mainly diffuses in ZIF-8 domains and/or that, in such conditions, the solution-diffusion model is not valid.

Figure 14b,c shows the solubility dependence on temperature for different gases in pure PSf and PSf/16 wt% ZIF-8. For H_2_, He, O_2_, and CO_2_, the solubility was slightly promoted by the filler presence and resulted in a decreasing function of temperature with a mild slope change due to ZIF-8 addition. Nitrogen solubility resulted to be almost independent on temperature at all filler loadings, while for CH_4_ solubility, the temperature dependence became less pronounced when high ZIF-8 loadings were reached.

Table S4 reports solubility and solubility selectivity values for five gases in membranes at 0%, 2%, 8%, and 16% by weight ZIF-8 loadings at 35 °C, 50 °C, and 65 °C.

### 3.7. Comparison with other MMMs

The present section focuses on comparing relative permeability and selectivity enhancements with respect to He/CO_2_ or H_2_/CO_2_ separation, obtained by adding ZIF-8 to different glassy polymers.

**Figure 15:**
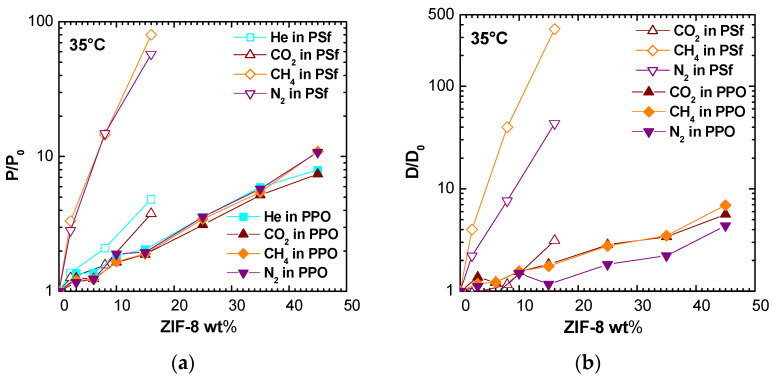
Comparison between (**a**) permeability and (**b**) diffusivity enhancement for various gases observed after addition of ZIF-8 to PSf (this work) and PPO ([19]) at 35 °C.

The data shown in Figure 15 refer to a previous work, focused on PPO/ZIF-8 mixed matrix membranes [19]. Considering the same filler loading, ZIF-8 addition produces a higher increase in permeability and diffusivity when added to PSf matrix. Furthermore, while the enhancement ratio is similar for all considered gases in case of PPO/ZIF-8 membranes, the permeability of N_2_ and CH_4_ is much more promoted with respect to CO_2_ and He when ZIF-8 addition in PSf is considered.

ZIF-8 addition to PPO increases diffusion coefficients of a factor lower than 10 at the highest loading for CO_2_, N_2_, and CH_4_, while ZIF-8 combination with PSf shifts the same factor to three for CO_2_, 43 for N_2_, and 360 for CH_4_ at 16% by weight of filler. This behavior could partially be explained by the intrinsically lower permeabilities in Polysulfone with respect to PPO, and the lower PSf fractional free volume (0.156 vs. 0.190). Thus, the addition of a relatively low-density filler as ZIF-8 (0.95 g/cm^3^ [48], 0.985 g/cm^3^ [19]) should promote gas transport properties to a greater relative extent in such polymer.

**Figure 16:**
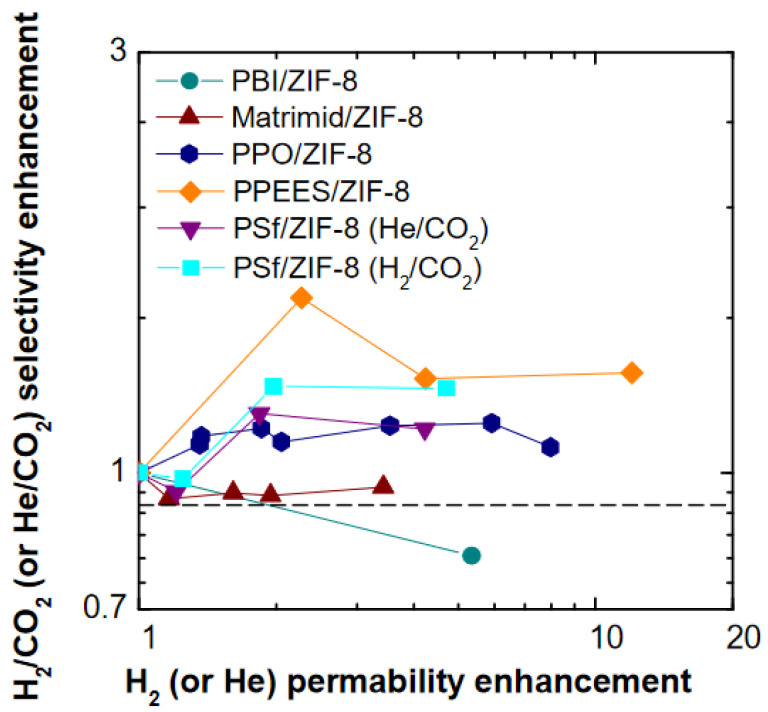
Comparison between He or H_2_ permeability enhancement and He/CO_2_ or H_2_/CO_2_ selectivity enhancement after addition of ZIF-8 to various size-selective membranes: PSf (this work), Matrimid^®^ [21], PPEES [64], PPO [19], and PBI [22].

The data shown in Figure 16 allow for the comparison between permeability and selectivity enhancement for He/CO_2_ or H_2_/CO_2_ pairs after ZIF-8 addition to different polymer matrices. The results obtained in this work for PSf are compared to other glassy polymers, such as Matrimid^®^ [21], PPEES [64], PPO [19], and PBI [22].

The permeability and selectivity enhancements obtained in this work are quite significant, considering a relatively low amount of ZIF-8 incorporated into PSf. Indeed, it can be seen that the values for PSf/16% ZIF-8 are similar to those obtained for PPEES/20% ZIF-8. These two polymers have a similar chemical structure, thus, the similarity in their behavior is quite expected. The permeability increase is comparable to PPO/35% ZIF-8 and PBI/10% ZIF-8; the selectivity improvement, however, is higher with respect to all other polymer matrices considered, except PPEES. Due to the intrinsically moderate selectivity of ZIF-8, remarkable enhancements in selectivity should not be expected when this filler is added to a size-selective polymer.

The results of this and previous papers indicate that ZIF-8 can be considered a permeability-enhancing additive in polymers for which this parameter is a limiting one, and for the separation of mixtures, like H_2_/CO_2_, for which the selectivity is not compromised by the presence of ZIF-8.

### 3.8. Permeability Modeling

As the properties of the MMMS inspected were noticed to be intermediate between those of the pure polymer and of the pure filler, we decided to check some of the data against a model valid for ideal composites, which assumes perfect adhesion between the phases and is valid below volume loadings of 20%. In particular, we chose to model the permeability and selectivity behavior using the Maxwell–Wagner–Sillars model for the H_2_/CO_2_ couple [65]. Under the assumption of dilute dispersion of ellipsoidal particles, fully oriented along the axis of the applied pressure difference, the permeability of composite membranes can be calculated as follows:

(10) $${\mathbb{P}}_{MMM}={\mathbb{P}}_{PSf}\;\frac{n\;{\mathbb{P}}_{ZIF-8}+\left(1-n\right)\;{\mathbb{P}}_{PSf}+\left(1-n\right)\;\varphi_{ZIF-8\;}\left({\mathbb{P}}_{ZIF-8}-{\mathbb{P}}_{PSf}\right)}{n\;{\mathbb{P}}_{ZIF-8}+\left(1-n\right)\;{\mathbb{P}}_{PSf}-n\;\varphi_{ZIF-8\;}\left({\mathbb{P}}_{ZIF-8}-{\mathbb{P}}_{PSf}\right)}\;$$

where $\varphi_{ZIF-8}$ is the volume fraction of the filler, and n is the shape factor of the filler.

Shape factor introduces information on geometrical orientation of the filler in the matrix. If spherical particles are considered, n=13, and Equation (10) simplifies into the well-known Maxwell equation. When the particles are elongated with the longest axis directed along the pressure gradient, 0<n<1/3, and the filler has higher influence on permeation properties of the composite membrane. Instead, when the shortest axis of an ellipsoidal particle is directed along the pressure gradient, the filler has lower influence on permeability and 1/3<n<1.

**Figure 17:**
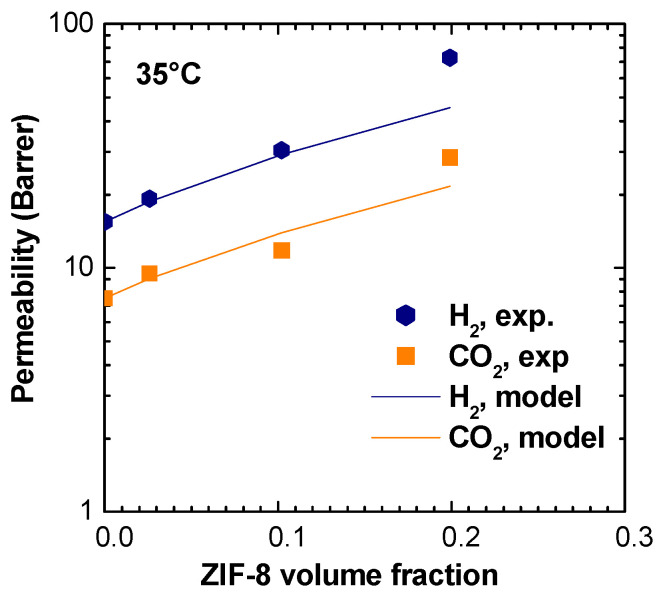
H_2_, CO_2_ permeabilities obtained with MWS model for PSf/ZIF-8 MMMs.

Being that shape factor is an adjustable parameter, the value that better describes our data was found to be equal to n=1/8. Using $\rho_{PSf}=1.22\;g/{\mathrm{cm}}^{3}$ and $\rho_{ZIF-8}=0.98\;g/{\mathrm{cm}}^{3}$, permeability values for H_2_ and CO_2_, together with the relative selectivity, can be modeled at 35 °C. Average values of ZIF-8 permeability, indicated in Table S1, were used. The modeling results are shown in Table 2 and in Figure 17.

The model describes well experimental results up to 10% in volume of ZIF-8 in the matrix, while above this loading, the actual permeability is higher than what predicted by the model for an “ideal” composite. This behavior is consistent with the low selectivities observed for certain gas couples in the MMMs containing the higher loadings of ZIF-8, which fall below the values of both the pure polymer and pure filler, indicating a non ideal behavior.

When applied to the entire range of gases inspected, the MWS model would predict a very weak effect of the gas nature and size on the permeability enhancement brought about by the addition of ZIF-8. In particular, the predicted permeability enhancement for the 16wt% loading lies between 2.90 and 2.98 for all the different gases, while experimentally higher values are measured, especially for the larger gases. The reason for this discrepancy between the model predictions and experimental observations lies partly in the basic model assumption of an ideal system with a non-interacting ensemble of filler particles, which may not be representative of our materials. Other aspects, such as the flexibility and breathability of the ZIF-8 structure, may also cause deviations from the model predictions as they are not accounted for by the model.

## 4. Conclusions

In this work, mixed matrix membranes based on PSf and variable amounts of ZIF-8 were fabricated with the aim to enhance the separation performance, particularly, with respect to the H_2_/CO_2_ mixture, and to provide a thorough characterization of the effect of gas size, temperature, and filler content on the transport behavior.

After morphological and density analysis, permeability tests were performed with six, gases, namely He, H_2_, CO_2_, O_2_, CH_4_, and N_2_, on membranes with ZIF-8 content up to 16 wt% at temperatures between 35 °C and 65 °C. The addition of ZIF-8 to Polysulfone produced a monotonous increase of permeability of all gases tested, with factors as high as 80. The trend was obeyed at all temperatures.

H_2_/CO_2_ and He/CO_2_ selectivities increased, to a smaller extent, up to 30–40% at 16 wt% ZIF-8 loading. The He/CO_2_ selectivity also increased with temperature. The ideal selectivity towards other gas mixtures, on the other hand, decreased after addition of ZIF-8, as expected due to the lower intrinsic selectivity of such filler with respect to the polymer for those mixtures.

The gas diffusivity also increased with the ZIF-8 content, for all gases and at each temperature. For the larger gases, such as N_2_ and CH_4_, the diffusivity enhancement was particularly high and led to the idea that those gases diffuse primarily in the ZIF-8 domains, whose pores were large enough to accommodate them, consistently with its flexible nature. This finding is consistent with the idea, reported in the literature, that the ZIF-8 pore size is around 4 Å, rather than 3.4 Å.

The energetic barrier to diffusion decreased, especially for bigger molecules, after addition of ZIF-8 to the PSf matrix, becoming almost zero in certain cases, due to the availability of more paths for gas transport in the presence of the filler particles. This trend is so marked that the activation energy of permeability assumes, at high filler loadings, the exothermic behavior typical of sorption, which makes ℙ decrease with temperature.

It is interesting to notice that the permeability of most mixed matrix membranes inspected was intermediate between that of the pure polymer and of the pure filler. We compared the data of H_2_ and CO_2_ transport with the Maxwell–Wagner–Sillars model, valid for ideal composite materials at moderate filler loadings. The model trend was obeyed for weight filler fractions lower than 10 wt%, while for higher loadings, a higher-than-ideal behavior was noticed, possibly due to the formation of a non-ideal interface with low-selectivity.

The present results, together with those obtained previously on polymers characterized by properties similar to PSf, indicate that the addition of ZIF-8 to such materials leads to mixed matrix membranes which can show remarkably higher permeability than the initial ones. However, the increase of selectivity achievable is not significant due to the intrinsically moderate or poor selectivity of ZIF-8 for different gas pairs, and this is consistent with literature results about polymer/ZIF-8 MMMs. Nonetheless, the relative enhancement of permeability and diffusivity obtained by adding ZIF-8 to PSf was higher than the one measured, at similar filler loadings, in other polymers such as PPO, probably because of its smaller initial free volume.

The results of the present and previous papers indicate that ZIF-8 is a valid possible permeability-enhancing additive to be used on those materials for which this parameter is critical and for those gas couples, such as H_2_/CO_2_ and He/CO_2_, where its addition is not detrimental to the selectivity.

## Figures and Tables

**Table 1:** Density values measured in pure PSf and MMMs and comparison with ideal additive values Equation (9).

	PSf	PSf/2% ZIF-8	PSf/8% ZIF-8	PSf/16% ZIF-8
$\rho_{MMM,exp}$	1.220 ± 0.013	1.195 ± 0.022	1.187 ± 0.018	1.164 ± 0.017
$\rho_{MMM,id}$	1.220	1.214	1.197	1.174

**Table 2:** H_2_, CO_2_ permeabilities, and H_2_/CO_2_ selectivity obtained with MWS model for PSf/ZIF-8 MMMs.

ZIF-8 Loading (wt %)	ϕ_ZIF-8_ (%)	H_2_ Permeability (Barrer)		CO_2_ Permeability (Barrer)		H_2_/CO_2_ Selectivity	
		Experimental	MWS Model	Experimental	MWS Model	Experimental	MWS Model
2	2.6	19.2 ± 0.9	18.7 ± 0.1	9.50 ± 0.45	9.0 ± 0.1	2.0	2.1
8	10.2	30.4 ± 3.8	29.3 ± 0.2	11.8 ± 1.4	14.0 ± 0.2	2.6	2.1
16	19.9	72.7 ± 11.9	45.6 ± 0.4	28.4 ± 4.5	21.8 ± 0.4	2.6	2.1

## Supplementary Materials

The following are available online at https://www.mdpi.com/article/10.3390/membranes11060427/s1. Section S1.1. Procedure for the calculation of mobility. Table S1. Permeability and selectivity values for PSf/ZIF-8 mixed matrix membranes. Table S2. Diffusivity and diffusivity-selectivity values for PSf/ZIF-8 mixed matrix membranes Table S3: Time lag values for PSf/ZIF-8 mixed matrix membranes. Table S4: Solubility coefficients and solubility selectivity values for PSf/ZIF-8 mixed matrix membranes. Figure S1: Permeability of various gases in PSf/ZIF-8 mixed matrix membranes as a function of ZIF-8 wt%: (a,b) at 50 °C; (c,d) at 65 °C. Figure S2: Permeability and selectivity in PSf/ZIF-8 mixed matrix membranes as a function of 1000/T: (a) Permeability in PSf/2% ZIF-8; (b) Permeability in PSf/8% ZIF-8. Figure S3: Diffusivity of various gases in PSf/ZIF-8 mixed matrix membranes as a function of ZIF-8 wt%: (a,b) at 50 °C; (c,d) at 65 °C. Figure S4: Diffusivity of various gases in PSf/ZIF-8 mixed matrix membranes as a function of 1000/T: (a) PSf/2% ZIF-8; (b) PSf/8% ZIF-8. Figure S5: Relative permeability and diffusivity increase of various gases in PSf/ZIF-8 mixed matrix membranes as a function of ZIF-8 wt%: (a,b) at 50 °C; (c,d) at 65 °C.
