# Supplementary material for: An Analysis of the Effect of ZIF-8 Addition on the Separation Properties of Polysulfone at Various Temperatures
Source: Membranes (Basel). 2021 Jun 4;11(6):427. doi: 10.3390/membranes11060427 (PMC8229596; doi:10.3390/membranes11060427)
Supplement: Supplementary file 1 [file membranes-11-00427-s001.zip › membranes-1206446-supplementary.pdf]

membranes

Supplementary Material

# An Analysis of the Effect of ZIF-8 Addition on the Separation Properties of Polysulfone at Various Temperatures

**Kseniya Papchenko [1], Giulio Risaliti [1], Matteo Ferroni,[2,3] Meganne Christian[2] and Maria Grazia De Angelis[1, 4*]**

[1] Department of Civil, Chemical Environmental and Materials Engineering, DICAM, University of Bologna, Via Terracini 28, 40131 Bologna, Italy. kseniya.papchenko2@unibo.it (K.P.); giulio.risaliti@studio.unibo.it (G.R.)
[2] CNR-IMM Section of Bologna, via Gobetti 101 - 40129 Bologna, Italy. christian@bo.imm.cnr.it (M.C.), ferroni@bo.imm.cnr.it (M.F.)
[3] Department of Civil, Environmental, Architectural Engineering and Mathematics, Università degli Studi di Brescia - Via Valotti, 9 - 25123 Brescia
[4] Institute for Materials and Processes, School of Engineering, University of Edinburgh, Sanderson Building, Robert Stevenson Road, EH9 3FB, UK.
* Correspondence: grazia.deangelis@ed.ac.uk (M.G.D.A.)

## S1.1. Procedure for the calculation of mobility

The diffusive flux $\mathcal{J}_i$ can be expressed either in terms of concentration gradient $\nabla\omega_i$ or in terms of penetrant chemical potential gradient $\nabla\mu_i$, that is the actual driving force for diffusion:

$$\mathcal{J}_i = -\mathfrak{D}_i \rho \nabla\omega_i = -\mathcal{L}_i \frac{\omega_i \rho}{RT} \nabla\mu_i \quad \text{(S1)}$$

In the first expression for the flux, originally given by Fick, the mutual diffusion coefficient $\mathfrak{D}_i$ appears, while in the second one the mobility, $\mathcal{L}_i$, or self-diffusion coefficient, is used. The mobility has a purely kinetic meaning and is related to the resistance of molecular motion in the solid mixture. [51–54] As a consequence of this equivalence, the coefficient $\mathfrak{D}_i$ can be rewritten as:

$$\mathfrak{D}_i = \frac{\mathcal{L}_i}{RT} \frac{\partial\mu_i}{\partial \ln\omega_i} \quad \text{(S2)}$$

If we assume the ideal gas model for the penetrant chemical potential in the gas phase, due to its low pressure, the chemical potential of gas $i$ can be related to the gas pressure and temperature and the respective pure component value, $\mu_{i,pure}$ at the same $T$ and $p$ as:

$$\mu_i = \mu_{i,pure}(T, p) + RT ln p_i \quad \text{(S3)}$$

So that the relation between the mutual diffusion coefficient and mobility can be rewritten as

$$\mathfrak{D}_i = \mathcal{L}_i \frac{\partial ln p_i}{\partial \ln\omega_i} \quad \text{(S4)}$$

Where the term multiplying the mobility is defined as $\alpha$

$$\alpha \equiv \frac{\partial ln p_i}{\partial \ln\omega_i} \quad \text{(S5)}$$

It is evident from its definition that $\alpha$ expresses a relation between the gas partial pressure in the external gaseous phase, and its corresponding concentration in the membrane polymeric phase, and is ultimately related to the solubility isotherm of the gas in the polymer membrane. This term is entirely thermodynamic in nature and is thus usually named the thermodynamic factor. Note that, based on the above equations the mutual

diffusivity can be decomposed into this purely thermodynamic factor and the purely kinetic mobility term, as follows:

$$\mathfrak{D}_i = \mathcal{L}_i \alpha \quad \text{(S6)}$$

Calculating the thermodynamic factor is possible either by using a thermodynamic model able to predict the gas solubility isotherm in the polymer membrane, or by taking experimental solubility isotherms for the specific gas-polymer system. In this paper we decided to follow this second route, as experimental isotherms for several gases in PSf are available in the literature. Such isotherms are normally given by points and one can approximate the differential relation defining $\alpha$ to the incremental ratio of $lnp_i$ vs. $\ln \omega_i$ in a sorption step going from an initial state *in* to a final state *fin*:

$$\alpha \cong \frac{\ln(\omega_i^{in}/\omega_i^{fin})}{\ln(p^{in}/p^{fin})} \quad \text{(S7)}$$

Here, sorption data at 35°C by Ghosal et al [6] were used in order to obtain $\alpha$ values for $O_2$, $N_2$, $CO_2$, and $CH_4$, estimated in the pressure range inspected in the permeation tests. Thermodynamic factor was found equal to 1.17 for $O_2$, 1.25 for $N_2$, 1.57 for $CH_4$, and 2.08 for $CO_2$, indicating a thermodynamic influence on diffusion coefficients for the considered gases. Using these values and the diffusivity obtained from this work, it was possible to obtain average mobility coefficients as the ratio between the mutual diffusion coefficients obtained *via* Equation (5) and the thermodynamic factor estimated from experimental literature data and Equation (S7).

**Table 1.** Permeability and selectivity values for PSf/ZIF-8 Mixed Matrix Membranes.

| | T (°C) | Permeability (Barrer[1]) | | | | | |
|---|---|---|---|---|---|---|---|
| | | $H_2$ | He | $O_2$ | $CO_2$ | $N_2$ | $CH_4$ |
| PSf | 35 | 15.4 ± 1.5 | 14.4 ± 0.8 | 1.50 ± 0.09 | 7.51 ± 0.45 | 0.26 ± 0.02 | 0.30 ± 0.02 |
| | 50 | 18.4 ± 1.8 | 17.5 ± 1.8 | 1.63 ± 0.17 | 7.78 ± 0.98 | 0.30 ± 0.04 | 0.37 ± 0.04 |
| | 65 | 21.9 ± 2.5 | 22.9 ± 2.7 | 2.41 ± 0.17 | 9.96 ± 1.10 | 0.51 ± 0.05 | 0.55 ± 0.05 |
| PSf/2% ZIF-8 | 35 | 19.2 ± 0.9 | 17.4 ± 0.9 | 2.18 ± 0.14 | 9.50 ± 0.45 | 0.74 ± 0.07 | 0.98 ± 0.12 |
| | 50 | 22.4 ± 1.7 | 21.4 ± 1.1 | 2.52 ± 0.14 | 10.5 ± 0.6 | 0.71 ± 0.08 | 1.09 ± 0.13 |
| | 65 | 25.9 ± 2.0 | 25.9 ± 1.3 | 2.90 ± 0.19 | 11.4 ± 0.7 | 0.76 ± 0.15 | 1.16 ± 0.05 |
| PSf/8% ZIF-8 | 35 | 30.4 ± 3.8 | 26.4 ± 4.3 | 4.65 ± 0.56 | 11.8 ± 1.4 | 3.88 ± 0.47 | 4.30 ± 0.58 |
| | 50 | 35.3 ± 4.4 | 31.7 ± 3.9 | 5.29 ± 0.69 | 13.4 ± 1.6 | 3.55 ± 0.45 | 4.56 ± 0.56 |
| | 65 | 37.7 ± 4.8 | 34.8 ± 4.4 | 4.79 ± 0.70 | 14.1 ± 2.0 | 3.02 ± 0.42 | 3.46 ± 0.48 |
| PSf/16% ZIF-8 | 35 | 72.7 ± 11.9 | 61.0 ± 11.4 | 16.2 ± 2.4 | 28.4 ± 4.5 | 15.0 ± 2.3 | 23.6 ± 3.7 |
| | 50 | 73.3 ± 11.4 | 59.7 ± 9.0 | 14.1 ± 2.1 | 25.5 ± 3.8 | 13.0 ± 2.8 | 16.1 ± 2.4 |
| | 65 | 71.9 ± 11.7 | 63.8 ± 9.7 | 12.4 ± 1.9 | 25.0 ± 4 | 9.39 ± 1.44 | 12.7 ± 1.9 |
| ZIF-8 [35,48,57–62] | 20–35 | 6691 ± 2923 | 3000 ± 1322 | 1600 ± 888 | 1640 ± 712 | 597 ± 221 | 542 ± 181 |

| | T (°C) | Selectivity | | | | | | |
|---|---|---|---|---|---|---|---|---|
| | | $H_2/CO_2$ | $He/CO_2$ | $H_2/CH_4$ | $CO_2/N_2$ | $CO_2/CH_4$ | $O_2/N_2$ | $CH_4/N_2$ |
| PSf | 35 | 2.06 ± 0.02 | 1.68 ± 0.01 | 52.4 ± 0.4 | 28.8 ± 0.2 | 25.5 ± 0.2 | 5.74 ± 0.04 | 1.13 ± 0.01 |
| | 50 | 2.36 ± 0.07 | 2.24 ± 0.07 | 49.8 ± 1.0 | 25.7 ± 0.7 | 21.1 ± 0.5 | 5.38 ± 0.1 | 1.22 ± 0.03 |
| | 65 | 2.20 ± 0.06 | 2.30 ± 0.06 | 39.6 ± 0.7 | 19.4 ± 0.4 | 18.0 ± 0.3 | 4.70 ± 0.1 | 1.08 ± 0.02 |
| PSf/2% ZIF-8 | 35 | 2.02 ± 0.01 | 1.83 ± 0.01 | 19.6 ± 0.4 | 12.8 ± 0.2 | 9.66 ± 0.20 | 2.95 ± 0.04 | 1.33 ± 0.03 |
| | 50 | 2.14 ± 0.01 | 2.04 ± 0.01 | 20.6 ± 0.5 | 14.7 ± 0.3 | 9.63 ± 0.20 | 3.55 ± 0.06 | 1.52 ± 0.07 |
| | 65 | 2.29 ± 0.02 | 2.28 ± 0.01 | 22.4 ± 0.1 | 14.9 ± 0.8 | 9.80 ± 0.05 | 3.80 ± 0.20 | 1.52 ± 0.07 |
| PSf/8% ZIF-8 | 35 | 2.58 ± 0.08 | 2.24 ± 0.08 | 7.07 ± 0.25 | 3.04 ± 0.09 | 2.74 ± 0.10 | 1.20 ± 0.04 | 1.11 ± 0.04 |
| | 50 | 2.63 ± 0.08 | 2.37 ± 0.07 | 7.07 ± 0.30 | 3.78 ± 0.10 | 2.94 ± 0.10 | 1.49 ± 0.05 | 1.28 ± 0.04 |
| | 65 | 2.67 ± 0.10 | 2.46 ± 0.09 | 10.9 ± 0.4 | 4.68 ± 0.20 | 4.09 ± 0.20 | 1.59 ± 0.06 | 1.14 ± 0.05 |

| | | | | | | | | |
|---|---|---|---|---|---|---|---|---|
| PSf/16% ZIF-8 | 35 | 2.57 ± 0.13 | 2.15 ± 0.12 | 3.09 ± 0.2 | 1.89 ± 0.10 | 1.20 ± 0.06 | 1.08 ± 0.05 | 1.57 ± 0.08 |
| | 50 | 2.88 ± 0.13 | 2.34 ± 0.11 | 4.55 ± 0.2 | 1.96 ± 0.20 | 1.58 ± 0.07 | 1.08 ± 0.09 | 1.24 ± 0.10 |
| | 65 | 2.88 ± 0.14 | 2.56 ± 0.12 | 5.64 ± 0.3 | 2.66 ± 0.10 | 1.96 ± 0.09 | 1.32 ± 0.06 | 1.36 ± 0.06 |
| ZIF-8 [35,48,57–62] | 20-35 | 4.1 ± 1.9 | 1.8 ± 0.9 | 12.3 ± 3.6 | 2.7 ± 0.9 | 3.0 ± 0.9 | 2.7 ± 1.1 | 0.9 ± 0.5 |

[1]Barrer: $10^{-10}$ $cm^3$ cm $cm^{-2}$ $s^{-1}$ $(cmHg)^{-1}$

**Table 2.** Diffusivity and diffusivity selectivity values for PSf/ZIF-8 Mixed Matrix Membranes.

| | T (°C) | **Diffusion Coefficient ($cm^2/s$)** x $10^7$ | | | | |
|---|---|---|---|---|---|---|
| | | $H_2$ | $O_2$ | $CO_2$ | $N_2$ | $CH_4$ |
| PSf | 35 | 3.79 ± 1.0 | 0.31 ± 0.05 | 0.10 ± 0.01 | 0.09 ± 0.01 | 0.015 ± 0.002 |
| | 50 | 9.68 ± 2.1 | 0.34 ± 0.07 | 0.14 ± 0.04 | 0.10 ± 0.02 | 0.04 ± 0.01 |
| | 65 | 13.3 ± 2.0 | 0.54 ± 0.16 | 0.24 ± 0.03 | 0.18 ± 0.03 | 0.09 ± 0.02 |
| PSf/2% ZIF-8 | 35 | 4.80 ± 1.0 | 0.21 ± 0.04 | 0.10 ± 0.01 | 0.20 ± 0.02 | 0.06 ± 0.01 |
| | 50 | 7.34 ± 1.5 | 0.42 ± 0.07 | 0.14 ± 0.02 | 0.19 ± 0.02 | 0.10 ± 0.02 |
| | 65 | 13.6 ± 3.7 | 0.59 ± 0.05 | 0.23 ± 0.02 | 0.23 ± 0.05 | 0.15 ± 0.02 |
| PSf/8% ZIF-8 | 35 | 8.63 ± 4.4 | 0.63 ± 0.16 | 0.12 ± 0.03 | 0.68 ± 0.23 | 0.59 ± 0.34 |
| | 50 | 18.6 ± 6.8 | 0.86 ± 0.24 | 0.18 ± 0.06 | 0.62 ± 0.21 | 0.83 ± 0.29 |
| | 65 | 24.3 ± 12.6 | 1.11 ± 0.39 | 0.24 ± 0.07 | 0.70 ± 0.20 | 0.81 ± 0.20 |
| PSf/16% ZIF-8 | 35 | 15.5 ± 8.4 | 1.37 ± 0.51 | 0.32 ± 0.10 | 3.84 ± 2.3 | 5.30 ± 1.7 |
| | 50 | 16.2 ± 6.8 | 1.50 ± 0.53 | 0.35 ± 0.10 | 4.02 ± 2.1 | 5.76 ± 1.7 |
| | 65 | 28.2 ± 13.5 | 1.38 ± 0.48 | 0.44 ± 0.14 | 4.18 ± 1.2 | 6.50 ± 1.9 |
| | **T (°C)** | **Diffusivity selectivity** | | | | |
| | | $H_2/CO_2$ | $H_2/N_2$ | $H_2/CH_4$ | $H_2/O_2$ | $CH_4/N_2$ |
| PSf | 35 | 36.8 ± 1.7 | 42.9 ± 2.4 | 258 ± 14 | 12.2 ± 1.0 | 0.17 ± 0.01 |
| | 50 | 70.1 ± 11.3 | 98.8 ± 8.6 | 258 ± 24 | 28.9 ± 2.5 | 0.38 ± 0.03 |
| | 65 | 56.6 ± 2.5 | 76.0 ± 4.1 | 142 ± 10 | 24.9 ± 3.5 | 0.54 ± 0.03 |
| PSf/2% ZIF-8 | 35 | 48.8 ± 1.3 | 24.5 ± 1.0 | 81.4 ± 5.9 | 23.4 ± 1.7 | 0.30 ± 0.01 |
| | 50 | 53.2 ± 2.2 | 38.4 ± 1.3 | 72.0 ± 6.4 | 17.4 ± 1.0 | 0.53 ± 0.02 |
| | 65 | 58.6 ± 2.2 | 58.6 ± 7.1 | 89.5 ± 6.5 | 23.2 ±0.7 | 0.66 ± 0.06 |
| PSf/8% ZIF-8 | 35 | 72.5 ± 15.2 | 12.8 ± 4.2 | 14.7 ± 13.6 | 13.7 ± 2.7 | 0.87 ± 0.30 |
| | 50 | 102 ± 23 | 30.0 ± 8.3 | 22.5 ± 6.6 | 21.6 ± 4.2 | 1.33 ± 0.37 |
| | 65 | 103 ± 28 | 34.6 ± 26.5 | 30.1 ± 6.2 | 21.9 ± 7.8 | 1.15 ± 0.66 |
| PSf/16% ZIF-8 | 35 | 48.3 ± 14.4 | 4.04 ± 4.3 | 2.92 ± 0.9 | 11.3 ± 4.4 | 1.38 ± 1.17 |
| | 50 | 46.3 ± 10.8 | 4.03 ± 2.8 | 2.81 ± 0.7 | 10.8 ± 3.4 | 1.43 ± 0.85 |
| | 65 | 64.7 ± 18.9 | 6.75 ± 1.7 | 4.34 ± 1.1 | 20.4 ± 6.6 | 1.56 ± 0.29 |

**Table S3.** Time lag values for PSf/ZIF-8 Mixed Matrix Membranes.

| | T (°C) | ℓ(μm) | Time lag θ (s) | | | | |
|---|---|---|---|---|---|---|---|
| | | | $H_2$ | $O_2$ | $CO_2$ | $N_2$ | $CH_4$ |
| PSf | 35 | 22.6 ± 1.3 | 2.3 ± 0.6 | 27.5 ± 4.8 | 83.0 ± 9.7 | 96.3 ± 13.0 | 578 ± 75 |
| | 50 | 20.8 ± 1.9 | 2.8 ± 0.6 | 21.6 ± 4.3 | 52.5 ± 15.2 | 73.6 ± 14.6 | 192 ± 40 |
| | 65 | 42.7 ± 2.8 | 1.9 ± 0.3 | 56.8 ± 16.7 | 129 ± 19.0 | 174 ± 27 | 323 ± 62 |
| PSf/2% ZIF-8 | 35 | 34.3 ± 1.4 | 4.8 ± 1.0 | 96.4 ± 17.7 | 199 ± 18 | 100 ± 12 | 334 ± 61 |
| | 50 | 34.3 ± 1.4 | 2.7 ± 0.5 | 46.4 ± 7.2 | 142 ± 18 | 103 ± 11 | 192 ± 40 |
| | 65 | 34.3 ± 1.4 | 2.0± 0.5 | 33.4 ± 3.0 | 84.4 ± 8.4 | 97.2 ± 22.2 | 130 ± 21 |
| PSf/8% ZIF-8 | 35 | 43.2 ± 5.0 | 3.8 ± 1.9 | 49.3 ± 12.2 | 261 ± 67 | 46.4 ± 15.8 | 56.8 ± 32.5 |
| | 50 | 43.2 ± 5.0 | 1.7 ± 0.6 | 36.1 ± 10.1 | 172 ± 52 | 50.4 ± 17.4 | 37.9 ± 13.5 |
| | 65 | 43.2 ± 5.0 | 1.3 ± 0.7 | 28.3 ± 10.0 | 132 ± 40 | 46.3 ± 24.4 | 38.5 ± 9.7 |
| PSf/16% ZIF-8 | 35 | 47.8 ± 6.9 | 2.6 ± 1.4 | 27.9 ± 10.3 | 119 ± 37 | 8.1 ± 4.8 | 7.2 ± 2.2 |
| | 50 | 47.8 ± 6.9 | 2.4 ± 1.0 | 25.4 ± 9.0 | 109 ± 32 | 9.8 ± 5.1 | 6.6 ± 2.0 |
| | 65 | 47.8 ± 6.9 | 1.4 ± 0.7 | 27.7 ± 9.6 | 88 ± 28.4 | 9.1 ± 2.7 | 5.9 ± 1.7 |

**Table S4.** Solubility coefficients and solubility selectivity values for PSf/ZIF-8 Mixed Matrix Membranes.

| | T (°C) | Solubility Coefficient S = P/D (cm³(STP)/cm³ pol atm) | | | | |
|---|---|---|---|---|---|---|
| | | $H_2$ | $O_2$ | $CO_2$ | $N_2$ | $CH_4$ |
| PSf | 35 | 0.32 ± 0.07 | 0.37 ± 0.04 | 5.57 ± 0.33 | 0.23 ± 0.02 | 1.52 ± 0.11 |
| | 50 | 0.14 ± 0.02 | 0.37 ± 0.04 | 4.30 ± 0.72 | 0.24 ± 0.03 | 0.75 ± 0.09 |
| | 65 | 0.13 ± 0.02 | 0.35 ± 0.05 | 3.22 ± 0.39 | 0.22 ± 0.02 | 0.45 ± 0.05 |
| PSf/2% ZIF-8 | 35 | 0.31 ± 0.05 | 0.81 ± 0.11 | 7.34 ± 0.36 | 0.29 ± 0.02 | 1.28 ± 0.27 |
| | 50 | 0.23 ± 0.03 | 0.45 ± 0.06 | 5.75 ± 0.44 | 0.29 ± 0.05 | 0.81 ± 0.08 |
| | 65 | 0.15 ± 0.04 | 0.38 ± 0.02 | 3.71 ± 0.17 | 0.28 ± 0.02 | 0.58 ± 0.07 |
| PSf/8% ZIF-8 | 35 | 0.28 ± 0.10 | 0.56 ± 0.07 | 7.52 ± 1.06 | 0.44 ± 0.10 | 0.60 ± 0.25 |
| | 50 | 0.15 ± 0.05 | 0.47 ± 0.07 | 5.62 ± 1.12 | 0.44 ± 0.10 | 0.42 ± 0.10 |
| | 65 | 0.12 ± 0.05 | 0.33 ± 0.07 | 4.57 ± 0.76 | 0.34 ± 0.13 | 0.33 ± 0.04 |
| PSf/16% ZIF-8 | 35 | 0.37 ± 0.15 | 0.90 ± 0.20 | 6.72 ± 1.07 | 0.31 ± 0.14 | 0.34 ± 0.05 |
| | 50 | 0.35 ± 0.09 | 0.72 ± 0.15 | 5.54 ± 0.83 | 0.25 ± 0.07 | 0.21 ± 0.03 |
| | 65 | 0.20 ± 0.07 | 0.69 ± 0.14 | 4.36 ± 0.80 | 0.17 ± 0.03 | 0.15 ± 0.02 |
| | T (°C) | Solubility selectivity | | | | |
| | | $CO_2/H_2$ | $O_2/H_2$ | $CO_2/N_2$ | $CO_2/CH_4$ | $O_2/N_2$ |
| PSf | 35 | 17.7 ± 1.0 | 1.17 ± 0.08 | 24.7 ± 0.3 | 3.65 ± 0.03 | 1.64 ± 0.02 |
| | 50 | 29.9 ± 1.2 | 2.58 ± 0.08 | 18.3 ± 0.6 | 5.75 ± 0.20 | 1.58 ± 0.04 |
| | 65 | 25.8 ± 0.8 | 2.76 ± 0.10 | 14.5 ± 0.2 | 7.17 ± 0.19 | 1.55 ± 0.03 |
| PSf/2% ZIF-8 | 35 | 23.9 ± 0.8 | 2.65 ± 0.13 | 25.6 ± 0.2 | 5.74 ± 0.34 | 2.84 ± 0.04 |
| | 50 | 24.7 ± 0.8 | 1.95 ± 0.07 | 20.1 ± 0.8 | 7.06 ± 0.13 | 1.59 ± 0.07 |
| | 65 | 25.1 ± 1.8 | 2.54 ± 0.20 | 13.1 ± 0.1 | 6.38 ± 0.13 | 1.33 ± 0.01 |
| PSf/8% ZIF-8 | 35 | 26.9 ± 5.6 | 2.00 ± 0.41 | 17.1 ± 1.5 | 12.6 ± 3.7 | 1.28 ± 0.11 |
| | 50 | 37.7 ± 8.1 | 3.13 ± 0.62 | 12.9 ± 1.3 | 13.3 ± 1.4 | 1.07 ± 0.09 |
| | 65 | 36.9 ± 8.4 | 2.67 ± 0.66 | 13.4 ± 3.5 | 14.0 ± 0.5 | 0.97 ± 0.27 |
| PSf/16% ZIF-8 | 35 | 18.1 ± 5.1 | 2.43 ± 0.75 | 21.5 ± 7.7 | 19.9 ± 1.0 | 2.88 ± 1.14 |
| | 50 | 15.9 ± 1.8 | 2.06 ± 0.27 | 22.0 ± 3.1 | 26.1 ± 1.3 | 2.84 ± 0.46 |
| | 65 | 21.9 ± 5.2 | 3.45 ± 0.84 | 25.5 ± 1.3 | 29.2 ± 1.5 | 4.01 ± 0.22 |

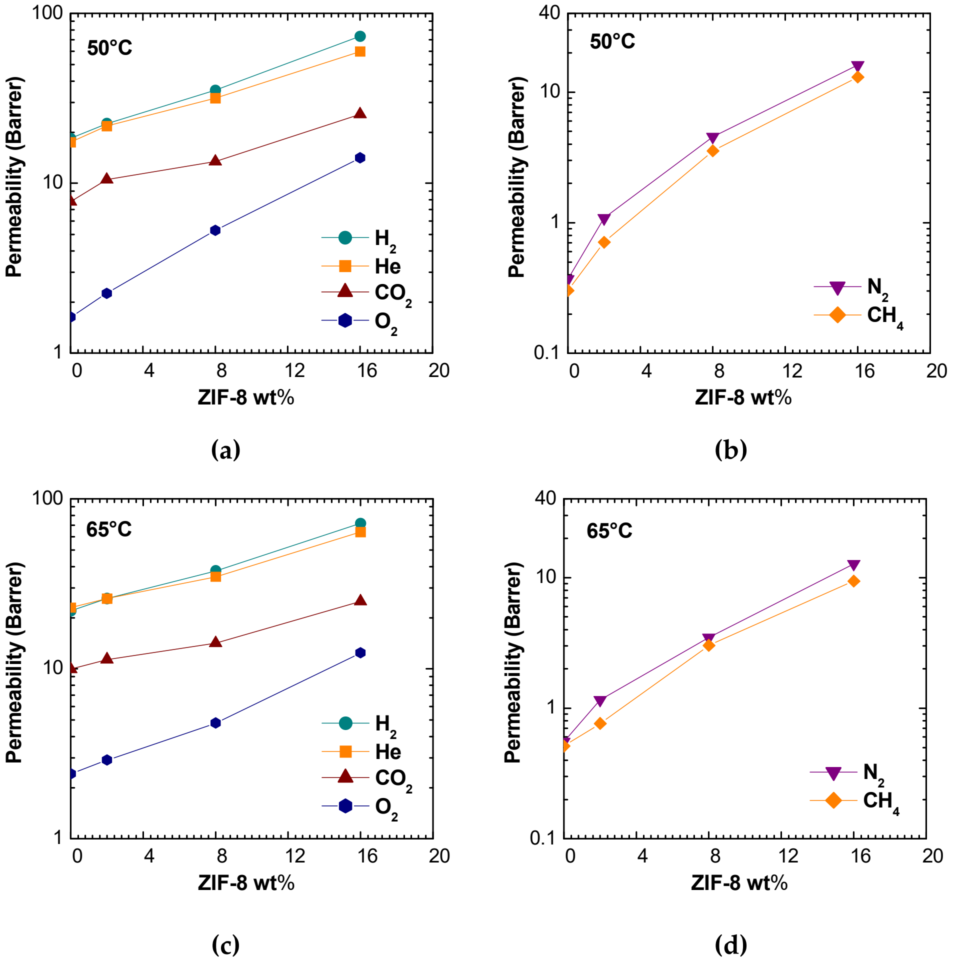


**Figure S1.** Permeability of various gases in PSf/ZIF-8 mixed matrix membranes as a function of ZIF-8 wt%: (**a**), (**b**) at 50°C; (**c**), (**d**) at 65°C.

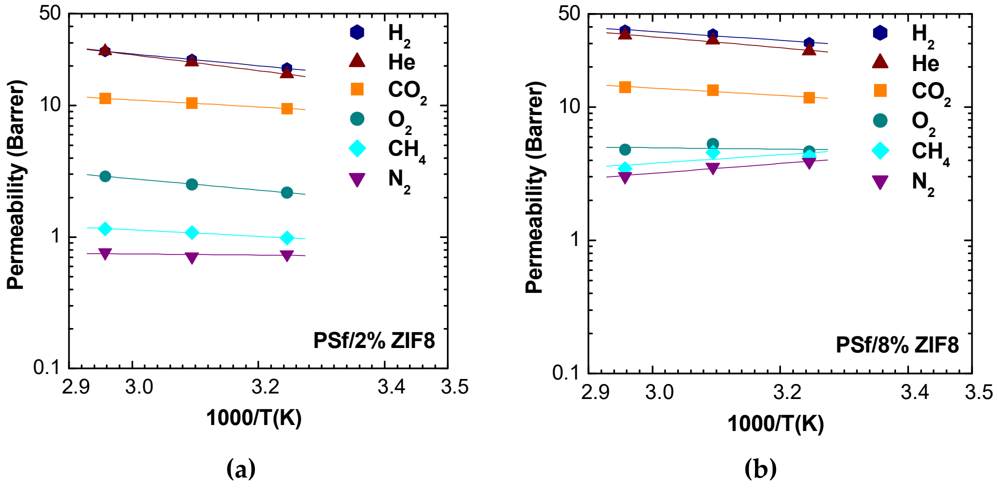


**Figure S2.** Permeability and selectivity in PSf/ZIF-8 mixed matrix membranes as a function of 1000/T: **(a)** Permeability in PSf/2% ZIF-8; (**b**) Permeability in PSf/8% ZIF-8.

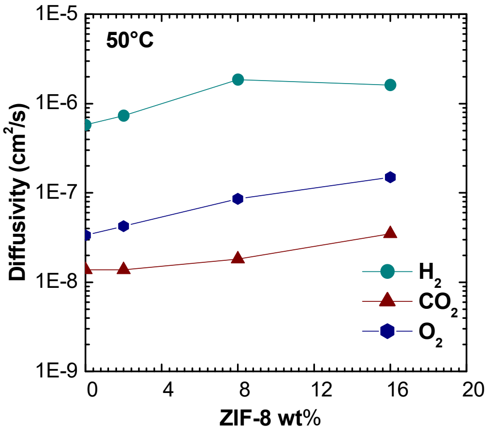


**(a)**

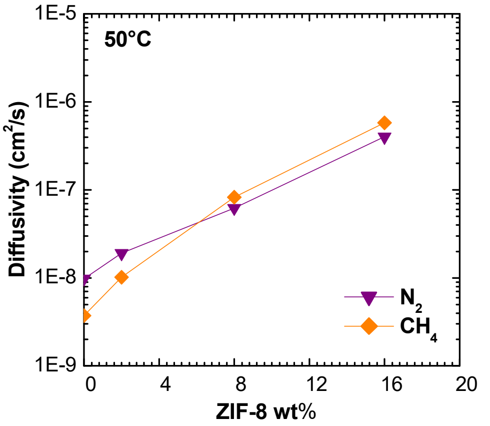


**(b)**

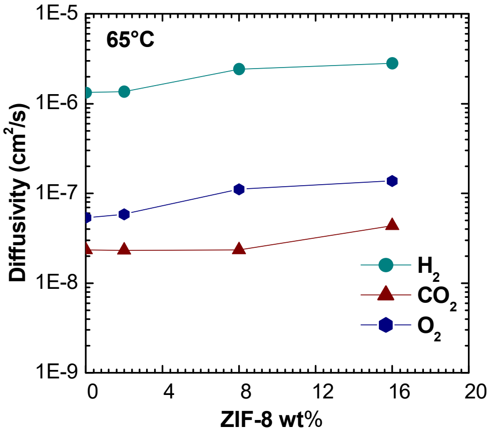


**(c)**

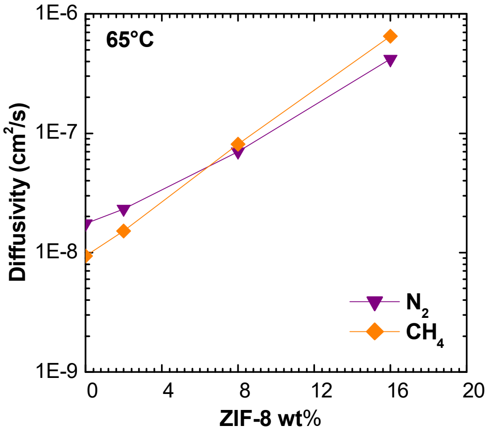


**(d)**

**Figure S3.** Diffusivity of various gases in PSf/ZIF-8 mixed matrix membranes as a function of ZIF-8 wt%: (**a**), (**b**) at 50°C; (**c**), (**d**) at 65°C.

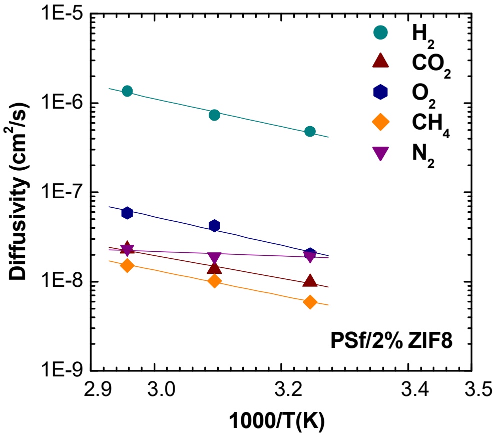


**(a)**

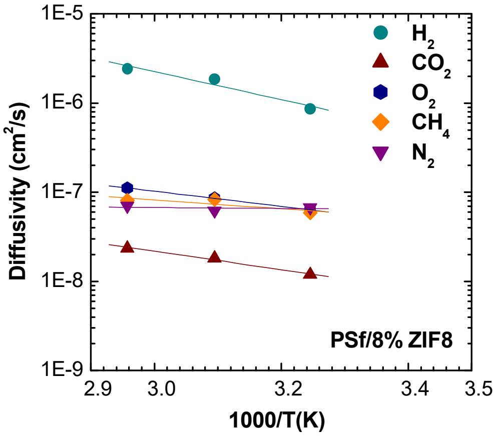


**(b)**

**Figure S4.** Diffusivity of various gases in PSf/ZIF-8 mixed matrix membranes as a function of 1000/T: (**a**) PSf/2% ZIF-8; (**b**) PSf/8% ZIF-8.

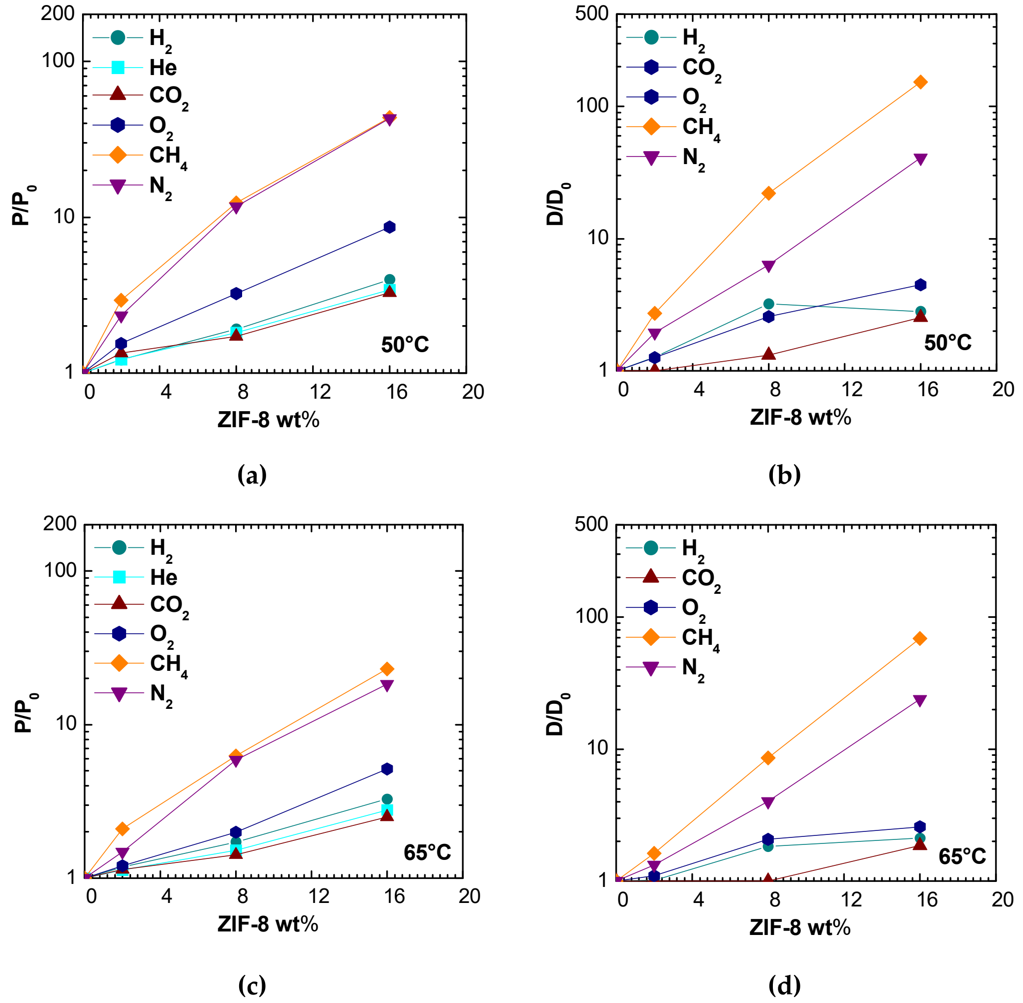


**Figure S5.** Relative permeability and diffusivity increase of various gases in PSf/ZIF-8 mixed matrix membranes as a function of ZIF-8 wt%: (**a**), (**b**) at 50 °C; (**c**), (**d**) at 65 °C.
